# Epitaxial Stabilization of Ultrasmall Cu Nanoparticles With High‐Energy {110} Facets on Ti_3_C_2_ MXene for Efficient CO_2_‐to‐Acetate Electrocatalysis

**DOI:** 10.1002/advs.75132

**Published:** 2026-04-02

**Authors:** Yan‐An Li, Jiapeng Huang, Yaohui Zhao, Junhao Lu, Yuan Ren, Zi‐Xin Ge, Qian Wang, Shangdong Ji, Yangzi Zheng, Chao Wu, Mingshang Jin

**Affiliations:** ^1^ Frontier Institute of Science and Technology and State Key Laboratory of Multiphase Flow in Power Engineering Xi'an Jiaotong University Xi'an Shaanxi China; ^2^ Interdisciplinary Research Center of Frontier Science and Technology Xi'an Jiaotong University Xi'an Shaanxi China

**Keywords:** acetate, CO_2_ reduction reaction, electrocatalysis, metal‐support electronic interaction, synergistic catalytic effect

## Abstract

The electrochemical CO_2_ reduction reaction (CO_2_RR) to multicarbon (C_2+_) products offers a promising route for sustainable carbon cycling and renewable energy storage. Copper (Cu)‐based nanocatalysts are indispensable for enabling C‐C coupling; however, conventional synthesis methods struggle to consistently produce and stabilize ultrasmall Cu(0) nanoparticles, which are prone to oxidation and aggregation, and fail to preserve their high‐energy facets critical for C_2+_ selectivity. In this work, we overcome these challenges by constructing a well‐defined Cu/Ti_3_C_2_ heterointerface that leverages a lattice matching mechanism. This approach not only stabilizes ultrasmall Cu(0) nanoparticles under ambient conditions but also promotes the preferential exposure and stabilization of high‐energy (110) facets. The resulting Cu/Ti_3_C_2_ composite catalyst exhibits exceptional performance in the CO_2_RR, achieving a total C_2_ Faradaic efficiency of 72.5%, with acetate alone reaching 42.5% at an industrially relevant current density of 235 mA·cm^−2^. Combined spectroscopic and computational studies reveal that the electronic metal‐support interaction and epitaxial growth are key to stabilizing the active structure, while the exposed Cu(110) facets lower the kinetic barriers for the critical C‐C coupling step toward acetate. This study underscores the vital importance of precise interfacial and crystallographic control in developing efficient and stable electrocatalysts for CO_2_ conversion.

## Graphical abstract

Cu/Ti_3_C_2_ heterostructure: Cu/Ti_3_C_2_ heterostructure achieves epitaxial stabilization of high‐energy Cu(110) facets through lattice matching and electronic interaction, enabling efficient CO_2_ electroreduction to acetate with 42.5% Faradaic efficiency at 235 mA·cm^−2^.

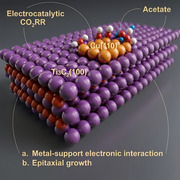

## 1 Introduction

By transforming atmospheric CO_2_ into carbon‐based chemicals and fuels, the electrocatalytic carbon dioxide reduction reaction (CO_2_RR) serves as a key technology for closing the carbon cycle and establishing a sustainable energy future [1, 2]. Among various catalysts, copper (Cu) stands out due to its unique ability to facilitate C‐C coupling, a crucial step for forming C_2+_ products like ethylene, ethanol, and acetate [3, 4, 5, 6]. Metallic copper Cu(0) has been identified as the active phase for CO_2_RR, yet sustaining it in an ultrasmall nanoparticle form is notoriously difficult [7, 8, 9]. However, conventional synthetic methods struggle to stabilize ultrasmall Cu nanoparticles (NPs) at ambient conditions, as they are prone to oxidation, aggregation, and structural degradation [10, 11]. This inherent instability leads to catalyst degradation and loss of active sites, making it exceptionally difficult to correlate initial catalyst structure with performance and thereby impeding rational catalyst design. Thus, the development of stable Cu(0) nanostructures and the establishment of clear structure‐activity relationships remain critical challenges in the field.

Recent advances highlight interface engineering as a powerful strategy for stabilizing metallic nanostructures [12, 13, 14]. For instance, in PdP@Pt core‐shell catalysts, strong Pt‐P interfacial interactions stabilize a single‐atom‐layer Pt structure, yielding a mass activity of 4.08 A mg_Pt_^−1^ and remarkable durability with only 8.9% activity decay after 50 000 cycles in the oxygen reduction reaction [15]. Similarly, in a tensile‐strained MoC@Pt catalyst, Pt‐Mo interfacial effects substantially enhance both activity and stability of the catalysts for the hydrogen evolution reaction [16]. Besides, constructing La inert oxide nano‐overlays on Pt/γ‐Mo_2_N catalyst protects the interfacial site, also significantly enhancing interfacial catalytic activity and stability, which activity decreased only 25% after 1300‐h reaction [17].

Cu NPs are notoriously susceptible to both oxidative degradation and facet reconstruction under CO_2_RR conditions, particularly the high‐energy facets essential for C‐C coupling [18]. Here, we address the dual challenges of stabilizing Cu NPs and preserving their high‐energy facets by constructing a Cu/Ti_3_C_2_ heterostructure. We demonstrate that the Ti_3_C_2_ support not only promotes the epitaxial growth of Cu(110) through lattice matching but also stabilizes these facets via metal‐support electronic interaction (MSEI), effectively suppressing surface reconstruction during electrocatalysis. The resulting catalyst exhibits exceptional performance, achieving a total C_2_ Faradaic efficiency (FE) of 72.5%, with acetate selectivity reaching 42.5% ± 2.1% at an industrially relevant current density of 235 mA cm^−2^, along with remarkable long‐term stability. Through a combination of advanced microscopy, in situ spectroscopy, and density functional theory (DFT) calculations, we confirm the predominant exposure and operational stability of the Cu(110) facets and elucidate the synergistic mechanism responsible for the high acetate selectivity. This work highlights the pivotal role of epitaxial stabilization and MSEI in the rational design of durable and selective Cu‐based electrocatalysts for sustainable CO_2_ conversion into value‐added multicarbon products.

## 2 Results and Discussion

### 2.1 Synthesis and Structural Characterization of the Cu/Ti_3_C_2_ Heterostructure

**FIGURE 1:**
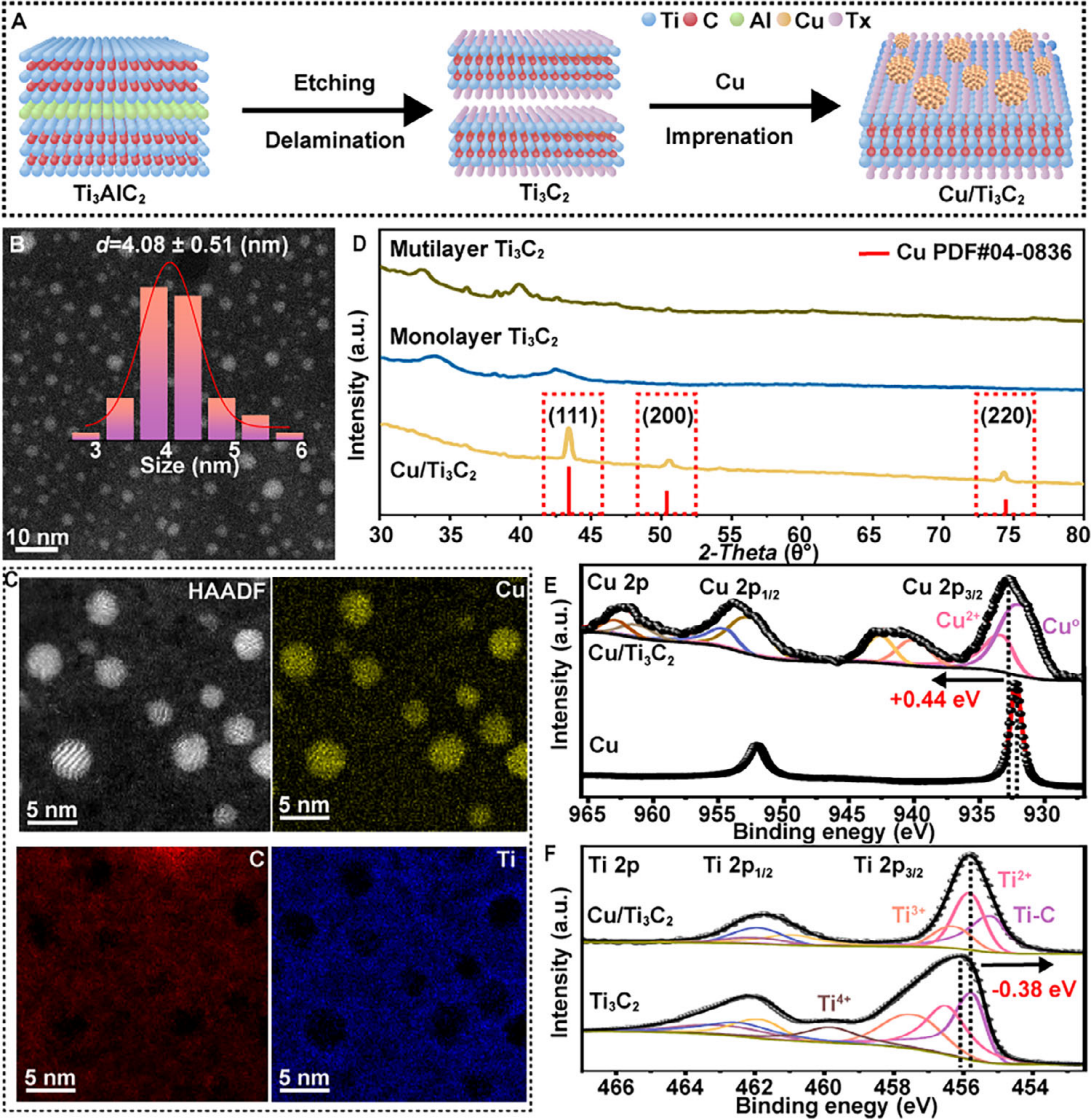
Synthesis and structural characterization of the Cu/Ti_3_C_2_ catalyst. (A) Schematic of the synthetic procedure. (B) HAADF‐STEM image and (C) corresponding elemental mapping, showing the uniform distribution of Cu NPs. (D) XRD patterns of multilayer Ti_3_C_2_, monolayer Ti_3_C_2_, and the Cu/Ti_3_C_2_ composite. High‐resolution XPS spectra of (E) Cu 2p and (F) Ti 2p.

The Cu/Ti_3_C_2_ catalyst was synthesized through a two‐step procedure (Figure 1A). First, monolayer Ti_3_C_2_ MXene nanosheets were obtained by selectively etching the Al layer from Ti_3_AlC_2_ using HF, followed by delamination via *N, N*‐dimethylformamide (DMF) intercalation and ultrasonic exfoliation [19, 20, 21]. Subsequently, ultrasmall Cu NPs were deposited onto the Ti_3_C_2_ support via a solution‐based reduction method. Scanning transmission electron microscopy (STEM) and high‐angle annular dark‐field STEM (HAADF‐STEM) images revealed uniformly dispersed ultrasmall Cu NPs on the Ti_3_C_2_ matrix, with an average diameter of ∼4 nm (Figure 1B). Elemental mapping and energy dispersive X‐ray spectroscopy (EDS) line scanning (Figure 1C; Figures S1 and S2) further confirmed the unique distribution of Ti, C, O, and Cu, demonstrating that Cu NPs are uniformly dispersed on the Ti_3_C_2_ support. X‐ray diffraction (XRD) as used to examine the crystalline structures of the materials (Figure 1D). The multilayer Ti_3_C_2_ sample (without intercalation) displayed a diffraction pattern in good agreement with previously reported profiles [22, 23, 24, 25]. After intercalation and exfoliation, the characteristic peak near 39° disappeared, confirming the successful delamination into monolayer Ti_3_C_2_ nanosheets [26, 27]. For the Cu/Ti_3_C_2_ composite, well‐defined diffraction peaks corresponding to metallic Cu were clearly observed, indicating the successful deposition of crystalline Cu NPs on the Ti_3_C_2_ support. The average crystalline size of Cu NPs was estimated to be ∼4 nm using the Scherrer equation (Table S1), which agreed well with the TEM observation. Furthermore, the relative intensity of the (220) reflection in the standard Cu PDF card (PDF#04‐0836) is 20%, whereas that of the Cu/Ti_3_C_2_ catalyst reaches 32.1%. This notable enhancement indicates a preferred <110> orientation in the catalyst. Notably, Cu NPs with a diameter of 4 nm are typically prone to oxidation under ambient conditions; the presence of metallic Cu peaks thus provides strong evidence for the effective stabilization of the Cu(0) state. X‐ray photoelectron spectroscopy (XPS) was employed to investigate the electronic interaction between Cu and Ti_3_C_2_. The survey spectrum confirmed the presence of Ti, C, O, and Cu in the Cu/Ti_3_C_2_ composite, while the F signal originates from the use of HF during the preparation of the Ti_3_C_2_ monolayer (Figure S3). High‐resolution XPS spectra revealed systematic binding energy shifts upon the formation of the heterostructure. As shown in Figure 1E,F, the binding energy of Cu 2p shifted toward higher binding energies by ∼0.44 eV, while the Ti 2p peaks shifted toward lower binding energies by ∼0.38 eV. These opposite shifts suggest electron transfer from Cu to Ti_3_C_2_, indicating MSEI with Cu acting as an electron donor and Ti_3_C_2_ as an acceptor. The satellite peaks indicate the presence of surface Cu^2+^ species arising from surface oxidation. However, given the 4 nm diameter of the Cu nanoparticles, approximately 40–50% of the Cu atoms are located at the surface. Consequently, even a single monolayer of surface oxide can generate a substantial Cu^2+^ XPS signal due to the high surface‐to‐bulk ratio [26, 27, 28, 29]. Importantly, our Cu/Ti_3_C_2_ sample exhibits a significantly lower Cu^2+^ content (36.5%), far below what would correspond to complete oxidation of the surface Cu atoms, demonstrating that the Ti_3_C_2_ support effectively protects these ultrasmall metallic particles from complete surface oxidation. The stability of this interaction was further demonstrated by the minimal changes in Cu LMM and Cu 2p spectra before and after catalytic tests (Figures S4 and S5).

**FIGURE 2:**
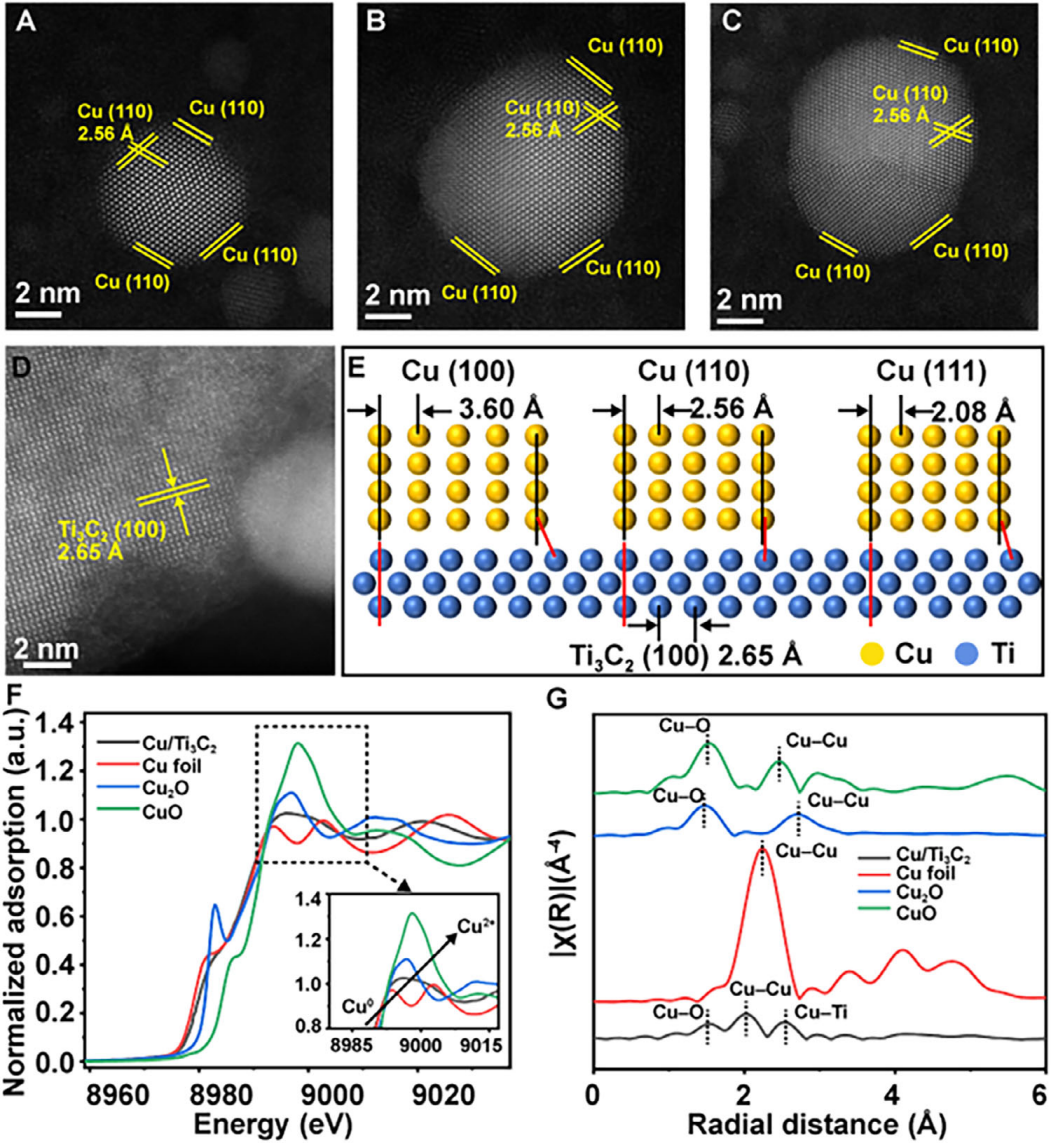
Atomic‐scale structure and local coordination of Cu in the Cu/Ti_3_C_2_ catalyst. (A–C) High‐resolution AC‐HAADF‐STEM images of Cu NPs. (D) AC‐HAADF‐STEM image of the Ti_3_C_2_ support. (E) Schematic model illustrating the epitaxial growth of Cu on Ti_3_C_2_, emphasizing the lattice matching at the interface. (F) Normalized Cu K‐edge XANES spectra and (G) Fourier‐transformed Cu K‐edge EXAFS spectra in R‐space for Cu/Ti_3_C_2_, with Cu foil, Cu_2_O, and CuO as references.

Figure 2A‐D displays aberration‐corrected high‐angle annular dark‐field scanning transmission electron microscopy (AC‐HAADF‐STEM) and high‐resolution TEM images of Cu/Ti_3_C_2_ composites. The Ti_3_C_2_ support exhibited lattice fringes corresponding to the {100} plane family (Figure S6), while the Cu NPs showed exposed {110} facets. Figure 2E presents an atomic model of the Cu/Ti_3_C_2_ heterostructure, illustrating the high degree of lattice matching between Cu(110) and Ti_3_C_2_(100). In heteroepitaxial systems, lattice matching (or controlled mismatch) introduces coherent interfacial strain at the Cu/Ti_3_C_2_ interface. The key lies in the significant lattice mismatch between Cu and the Ti_3_C_2_ substrate. This substantial mismatch creates a high energy barrier for the lateral spreading of Cu atoms across the Ti_3_C_2_ surface. Due to the large lattice mismatch between Cu and Ti_3_C_2_, layer‐by‐layer growth becomes energetically unfavorable because the strain energy accumulated in a continuous film would be high [28, 29, 30, 31]. To minimize the total system energy, Cu atoms no longer favor complete surface wetting. Instead, they preferentially nucleate at surface defect sites, such as steps, vacancies, or functional groups on Ti_3_C_2_, and grow vertically into 3D island structures. This Volmer‐Weber growth mode naturally limits lateral expansion and constrains particle size, thereby suppressing excessive nanoparticle growth, which is consistent with previous reports [32, 33, 34]. The preferential orientation of Cu nanoparticles is governed by the lattice mismatch between Cu and the Ti_3_C_2_ substrate. Specifically, the small lattice mismatch between Cu(110) and Ti_3_C_2_(100) makes this orientation energetically favorable, thereby stabilizing the exposed high‐energy Cu(110) facets. Direct evidence for this orientation relationship is provided by high‐resolution HAADF‐STEM imaging of the Cu/Ti_3_C_2_ interface (Figure S7), which clearly reveals that the Cu(110) plane is aligned parallel to the Ti_3_C_2_(100) surface. These observations are consistent with established principles of heteroepitaxial growth on substrates with lattice mismatch [35, 36].

Electron energy loss spectroscopy (EELS) analysis confirmed that the NPs existed primarily in the metallic Cu(0) state (Figure S8). Further investigation using X‐ray absorption spectroscopy provided additional evidence for the local coordination environment of Cu. The X‐ray absorption near‐edge structure (XANES) spectra showed an absorption edge position for Cu/Ti_3_C_2_ similar to that of Cu foil (Figure 2F), indicating a metallic state of Cu. Fourier‐transformed extended X‐ray absorption fine structure (FT‐EXAFS) in R space (Figure 2G; Figure S9) displayed a primary peak at ∼1.54 Å corresponding to Cu−O coordination, along with a prominent peak at ∼2.03 Å corresponding to the Cu−Cu metallic bond. A distinct scattering peak at ∼2.53 Å provided evidence for Cu−Ti interaction, confirming interfacial contact between Cu NPs and the Ti_3_C_2_ support [37, 38, 39]. Wavelet transform analysis further distinguished the contributions of Cu‐Cu and Cu‐Ti bonds (Figure S10), visually confirming the coexistence of metallic Cu NPs and Cu‐Ti interaction in the Cu/Ti_3_C_2_ composite. EXAFS fitting analysis was conducted to quantify the local coordination environment of Cu in the Cu/Ti_3_C_2_ catalyst (Figure S9). The Cu‐Cu coordination number (CN) of 3.7 is significantly lower than that of the bulk Cu (CN = 12), indicating that a substantial fraction of Cu atoms is not coordinated to other Cu atoms but directly to the Ti_3_C_2_ support. The Cu‐Ti CN of 3.6 ± 0.2 approaches the Cu‐Cu CN (3.7), further indicating that nearly 50% of the Cu coordination sites are occupied by Ti atoms from the support. This extensive interfacial bonding implies that Cu atoms directly interacting with Ti_3_C_2_ are physically and electronically stabilized against oxidation. The very low Cu‐O CN of 0.7 ± 0.1 demonstrates that oxidation is severely limited. The coexistence of dominant Cu‐Cu and Cu‐Ti contributions with a weak Cu‐O contribution suggests only limited surface oxidation of the Cu nanoparticles, rather than the formation of bulk CuO or Cu_2_O phases. The significantly lower Cu‐Cu coordination number compared to that of Cu foil indicates the exposure of Cu(110) facets that are structurally more open than other low‐index facets in Cu NPs (Table S2) [40].

### 2.2 Electrochemical CO_2_RR Performance

**FIGURE 3:**
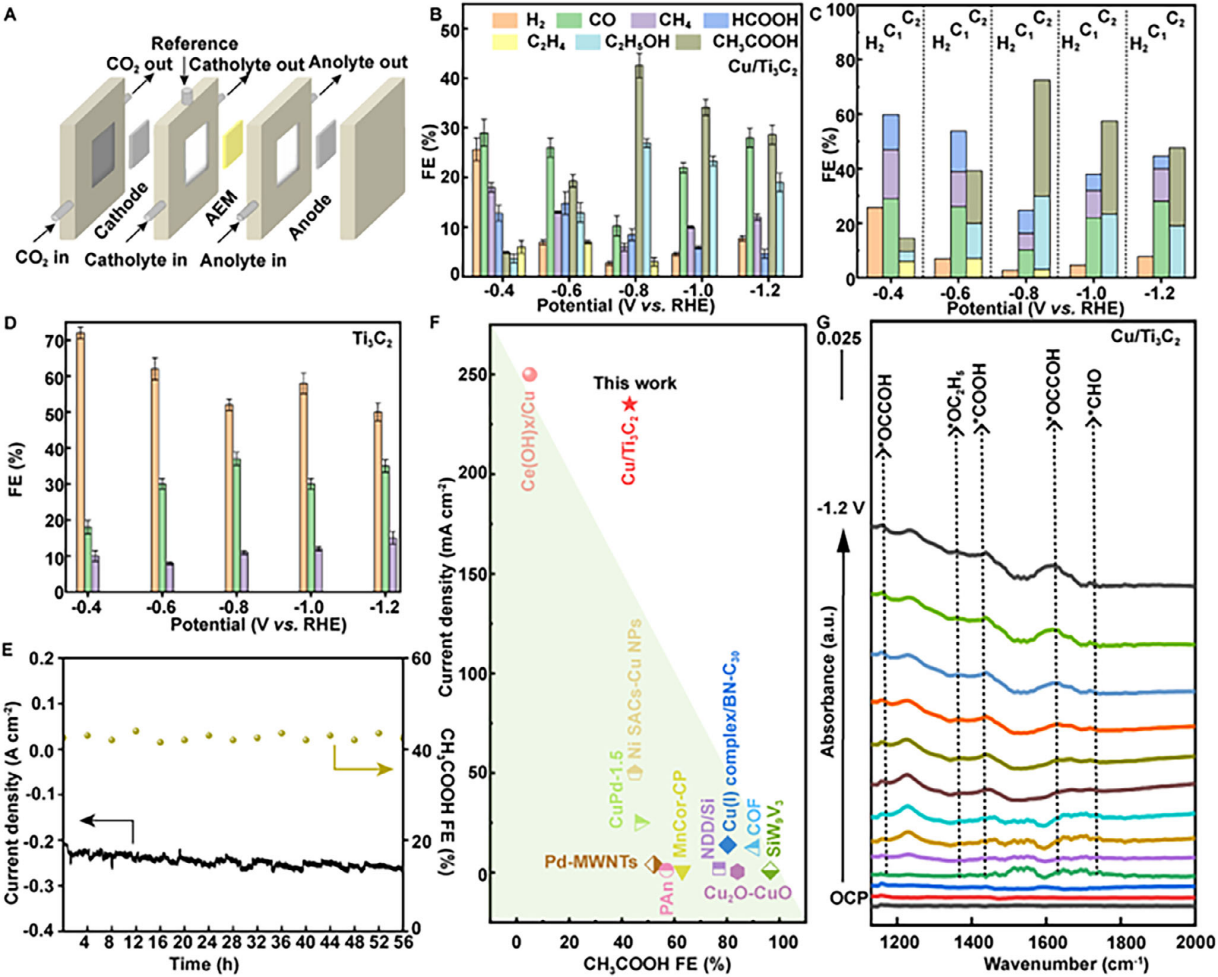
Electrocatalytic CO_2_ reduction performance of Cu/Ti_3_C_2_ in a flow cell system. (A) Schematic diagram of the flow cell setup. (B) Potential‐dependent product distribution and (C) corresponding Faradaic efficiencies for Cu/Ti_3_C_2_. (D) Product distribution for Ti_3_C_2_ alone at various potentials. (E) Long‐term stability test of Cu/Ti_3_C_2_ at −0.8 V *vs*. RHE. (F) Comparison of acetate Faradaic efficiency versus current density between this work (star) and previously reported Cu‐based catalysts. (G) In situ ATR‐SEIRAS spectra collected during CO_2_RR on Cu/Ti_3_C_2_ from −0.1 to −1.2 V *vs*. RHE, showing the evolution of key reaction intermediates.

The CO_2_ reduction performance of the Cu/Ti_3_C_2_ catalyst was first evaluated by linear sweep voltammetry (LSV) in a three‐electrode H‐cell using 1.0 M KOH saturated with Ar or CO_2_. As shown in Figure S11, a significantly enhanced current density was observed under a CO_2_ atmosphere relative to an Ar atmosphere, confirming the occurrence of the electrocatalytic CO_2_ reduction reaction. To better evaluate the catalytic performance under practical conditions, the CO_2_RR measurements were subsequently conducted in a flow‐cell reactor (schematic in Figure 3A). In this configuration, the Cu/Ti_3_C_2_ catalyst achieved a substantially higher total current density (Figure S12). Gaseous and liquid products were quantified using online gas chromatography (GC) and ^1^H nuclear magnetic resonance (NMR) spectroscopy, respectively. As shown in Figure 3B, the Cu/Ti_3_C_2_ catalyst produced a range of products, including H_2_, CO, CH_4_, and C_2_H_4_ in the gas phase, along with formic acid, ethanol, and acetate in the liquid phase (Figure S13). The product distribution and corresponding Faradaic efficiency (FE) were strongly dependent on the applied potential (Figure 3C). Notably, at −0.8 V *vs*. RHE, the catalyst exhibited exceptional performance, achieving a FE of 42.5% for acetate and a total C_2_ product selectivity of 72.5%. The total current density at this potential reached 235 mA cm^−2^, indicating high overall activity while maintaining excellent acetate selectivity. This combination of high current density and selectivity surpasses most previously reported catalysts (Table S3). The potential‐dependent profile revealed that acetate formation was optimized at −0.8 V, with efficiency decreasing at both higher and lower potentials (Figure 3B). Control experiments using Ti_3_C_2_ alone under identical conditions produced only H_2_, CO, and CH_4_, with no C_2_ products detected (Figure 3D). This stark contrast highlights the essential role of Cu NPs in enabling C‐C coupling and facilitating the formation of multi‐carbon products. Notably, even on the same Ti_3_C_2_ support, previous studies have reported no significant acetate selectivity for Cu nanoparticles in the absence of specific facet control, further underscoring that the exposed Cu(110) facets, rather than the support or particle size alone (Table S4) [41, 42, 43, 44, 45, 46, 47, 48]. Furthermore, our Cu/Ti_3_C_2_ catalyst retains its high selectivity toward acetate even in an H‐cell configuration electrolyzer (Figure S14).

The operational stability of Cu/Ti_3_C_2_ was assessed through a continuous 55‐hour test at −0.8 V vs. RHE. The catalyst maintained stable performance with an average acetate FE of approximately 42% and no significant activity decay (Figure 3E). Post‐reaction characterization confirmed that the Cu NPs remained well‐dispersed on the Ti_3_C_2_ support (Figures S15 and S16), and the crystal structure was largely preserved (Figure S17), demonstrating the structural robustness of the heterostructure under prolonged operation. The 4 nm Cu nanoparticles retain their size and dispersion on the Ti_3_C_2_ support after 55 h of continuous CO_2_ reduction at −0.8 V vs. RHE. No significant particle growth or sintering was observed, confirming the stabilizing effect of the strong Cu‐Ti_3_C_2_ interaction. The Cu/Ti_3_C_2_ interfacial structure remains intact, with no evidence of delamination or phase separation. Besides, the inductively coupled plasma optical emission spectroscopy (ICP‐MS) indicated that a negligible loss of Cu and Ti confirms that no significant Cu leaching occurs during electrocatalysis (Table S5). This excellent stability is mainly attributed to the strong interfacial interaction between Cu nanoparticles and Ti_3_C_2_ support, which effectively anchors the Cu nanoparticles against dissolution. These results demonstrate that the Cu/Ti_3_C_2_ catalyst exhibits excellent recyclability and durability, highlighting its potential for industrial application. When compared with previously reported Cu‐based and MXene‐supported catalysts, the Cu/Ti_3_C_2_ composite shows superior performance in both acetate selectivity and operating current density (Figure 3F), positioning it as a highly promising candidate for practical CO_2_ electroreduction applications.

To elucidate the origin of the high acetate selectivity, the CO_2_RR process on the Cu/Ti_3_C_2_ catalyst was monitored using in situ attenuated total reflection surface‐enhanced infrared absorption spectroscopy (ATR‐SEIRAS) and *operando* Raman spectroscopy. At the open‐circuit potential (OCP), no observed intermediates were detected, indicating that the formation of reactive species is both potential‐ and current‐dependent. When the applied potential was scanned from −0.1 to −1.2 V *vs*. RHE, multiple characteristic infrared bands emerged in the ATR‐SEIRAS spectra (Figure 3G). The bands observed at 1170, 1370, and 1720 cm^−1^ were assigned to *OCCOH, *OC_2_H_5_, and *CHO, respectively, all of which are recognized as crucial surface species involved in C‐C coupling during CO_2_RR [49, 50]. In particular, *OCCOH is widely established as a common intermediate to various C_2_ products, including C_2_H_4_, C_2_H_5_OH, and CH_3_COOH [51, 52, 53, 54, 55, 56, 57, 58]. The reaction pathways diverge from the *OCCOH intermediate. The route leading to C_2_H_4_ involves hydrogenation to form *HOCCOH, while the pathways for C_2_H_5_OH and CH_3_COOH formation initially follow a common sequence consisting of *OCCOH, *OCC, and *OCCH. A key branching point occurs at the *OCCH intermediate, where subsequent hydrogenation steps determine the final product distribution. The transformation of *OCCH into *OCHCH leads to ethanol, whereas progression along the acetate pathway proceeds through the formation of *OCCH_2_ [59]. These mainly intermediates, involved in electrocatalytic CO_2_RR, were detected via in situ ATR‐SEIRAS (Figure 3G; Figure S18). The detection of this comprehensive set of C_2_‐related intermediates, especially those specific to the acetate‐forming pathway, provides direct spectroscopic evidence that the Cu/Ti_3_C_2_ catalyst actively promotes C‐C coupling and selectively leads the reaction pathway toward acetate.

Based on the experimental and spectroscopic evidence, we propose that acetate formation on the Cu/Ti_3_C_2_ catalyst proceeds via a synergistic mechanism between the Cu active‐site and the Ti_3_C_2_ support. The Ti_3_C_2_ support acts as a cocatalyst that facilitates the initial activation of CO_2_, leading to the generation of *CO and *H species [54, 55]. The reaction mechanism involves several key steps. First, *CO adsorbed on the Cu(110) active sites reacts with atomic hydrogen supplied by the Ti_3_C_2_ support to form *COH. This *COH intermediate then couples with another *CO molecule, either from the support or adjacent sites, forming the critical *OCCOH dimer [56, 57]. This C_2_ intermediate undergoes sequential reduction through *OCC and *OCCH species. The selectivity toward acetate arises from the different pathways of hydrogenation at the *OCCH intermediate, where hydrogenation toward *OCCH_2_ rather than *OCHCH would lead to the formation of CH_3_COOH. *Operando* Raman spectroscopy was performed to further validate the formation mechanism of acetate by probing adsorbed intermediates during the reaction. The spectra of Cu/Ti_3_C_2_ exhibited a distinct band near 1900 cm^−1^, attributed to bridge‐absorbed *CO species (Figure S19) [58, 59]. In contrast, Ti_3_C_2_ support along displayed only linearly adsorbed *CO in the 2100–2150 cm^−1^ region (Figure S20). The presence of bridge‐bound *CO on Cu/Ti_3_C_2_ indicates a favorable configuration for C‐C coupling, consistent with a mechanism where *CO on Cu active sites interacts with *H supplied by the Ti_3_C_2_ support, leading to *OCCOH formation and subsequent C_2_ product generation.

### 2.3 Mechanistic Insights

**FIGURE 4:**
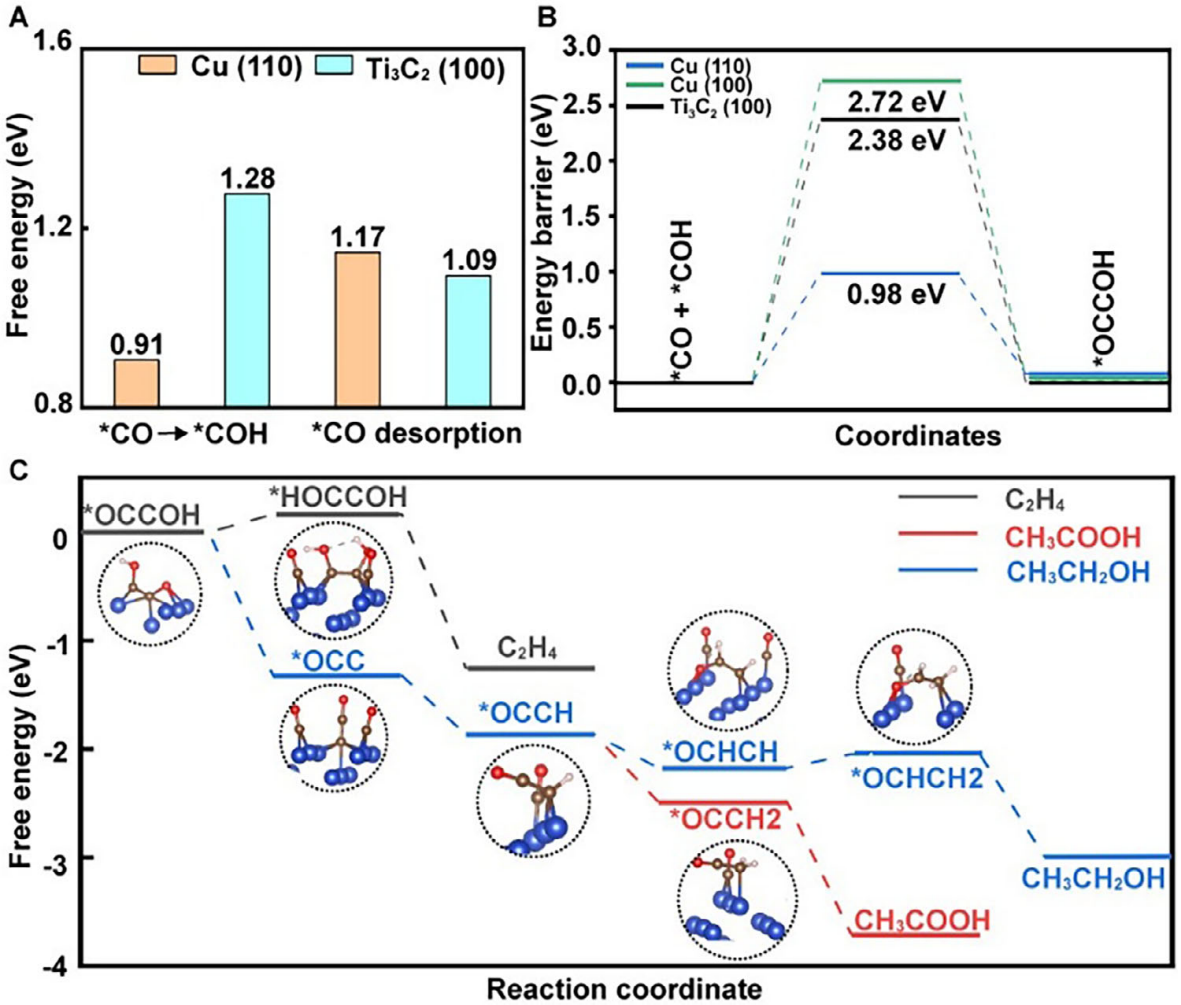
DFT‐calculated reaction pathways and energetics for CO_2_ reduction on Cu/Ti_3_C_2_. (A) Gibbs free energy changes for *CO hydrogenation to *COH and *CO desorption on Cu(110) and Ti_3_C_2_(100) surfaces. (B) Reaction free energies for *OCCOH formation on different surfaces. Dotted lines serve as visual guides. (C) Free energy profiles for the formation of C_2_H_4_, C_2_H_5_OH, and CH_3_COOH from key intermediates on Cu/Ti_3_C_2_. Insets depict the optimized atomic configurations of relevant intermediates, with Cu, O, C, and H atoms represented by blue, red, brown, and white spheres, respectively.

Density functional theory (DFT) calculations were performed to elucidate the origin of the high CO_2_RR selectivity toward acetate on the Cu/Ti_3_C_2_ catalyst. A structural model of the Cu/Ti_3_C_2_ interface was constructed based on experimental evidence, with Cu(110) and Ti_3_C_2_(100) facets selected as the representative surfaces (Figures S21 and S22). First, the calculations revealed that the formation energy of *COH from *CO hydrogenation is more favorable on Cu/Ti_3_C_2_ than on Ti_3_C_2_, whereas *CO desorption is energetically preferred on Ti_3_C_2_ (Figure 4A). Raman spectroscopy and in situ ATR‐SEIRAS (Figure 3G) detected *CO and *OCCOH intermediates on Cu/Ti_3_C_2_, indicating that C‐C coupling proceeds via the pathway involving *CO and *COH to form *OCCOH, consistent with literature reports [56, 57]. Therefore, we further performed DFT calculations comparing the coupling barriers on different surfaces based on the *OCCOH pathway. As shown in Figure 4B, the energy barrier for this C─C coupling step on Cu(110) is 0.98 eV, substantially lower than on Cu(100) (2.72 eV). This difference indicates a kinetically more favorable C‐C coupling on Cu(110). We also examined the catalytic performance of Ti_3_C_2_(100) support, which exhibits an energy barrier of 2.38 eV, much higher than on Cu(110), suggesting that C‐C coupling would preferentially occur on the Cu(110) surface rather than on the support.

These results align well with experimental observations, supporting a synergistic mechanism in which Cu active sites and the Ti_3_C_2_ support jointly facilitate CO_2_ reduction to C_2_ products. To further understand the selectivity toward acetate, the reaction pathway was traced from *OCCOH, a key C_2_ intermediate widely reported in the literature [60, 61, 62, 63, 64, 65]. The proposed mechanism involves sequential reduction and protonation steps of *OCCOH to *OCC to *OCCH to *OCCH_2_ to CH_3_COOH. The unique selectivity toward acetate on Cu(110) originates from its distinct surface geometry and electronic structure. The open stepped configuration of Cu(110) stabilizes oxygen‐bound intermediates such as *OCC, *OCCH, and *OCCH_2_, facilitating sequential protonation steps that ultimately yield acetic acid. Furthermore, our DFT calculations indicate that high CO coverage on Cu(110), achieved through strong *CO binding and the promotional effect of the Ti_3_C_2_ support, favors *CO protonation by surface *H to form *COH. This *COH intermediate subsequently couples with *CO to form the C‐C bond, opening an acetate‐specific pathway distinct from the *CO dimerization route that typically leads to ethylene and ethanol. Moreover, in alkaline electrolyte, water serves as the proton source, with the reaction potentially proceeding through a ketene (CH_2_‐CO) intermediate that undergoes hydrolysis to acetic acid [51, 64]. The free energy diagram in Figure 4C indicates that the pathway from *OCCOH to acetate is thermodynamically more feasible than those leading to C_2_H_4_ or C_2_H_5_OH. Although the *OCCH intermediate is common to both ethanol and acetate pathways, further hydrogenation toward *OCCH_2_ is energetically favored over the route to *OCHCH on Cu/Ti_3_C_2_, rationalizing the high selectivity to acetate. Collectively, the DFT calculations, together with in situ ATR‐SEIRAS and *operando* Raman spectroscopy, validate the proposed synergistic catalysis mechanism. In this mechanism, *CO adsorbed on Cu sites reacts with *H supplied by Ti_3_C_2_ to form *COH, which subsequently couples with another *CO to generate *OCCOH on the Cu(110) facets.

## 3 Conclusions

In summary, this study demonstrates the design of a Cu/Ti_3_C_2_ composite catalyst for efficient CO_2_ electroreduction to acetate through epitaxial stabilization driven by metal–support electronic interaction. The Ti_3_C_2_ support not only stabilizes ultrasmall metallic Cu nanoparticles against oxidation but also promotes and preserves the high‑energy (110) facets via lattice matching and charge transfer. Combined mechanistic and computational analyses reveal a synergistic catalytic effect wherein the Ti_3_C_2_ support enriches *CO intermediates, while the exposed Cu(110) facets significantly lower the energy barriers for *CO hydrogenation and C‐C coupling. This well‑designed interface enables outstanding CO_2_‑to‑C_2_ performance, achieving a total FE of 72.5% and a current density of 235 mA·cm^−2^. More notably, the catalyst exhibits excellent stability, maintaining an acetate FE of 42.5% over 55 h of continuous operation at industrial‐scale current density. These findings provide a rational catalyst design strategy that couples facet engineering with interfacial stabilization, offering a promising route toward the sustainable electrochemical synthesis of multicarbon chemicals from CO_2_.

## Conflicts of Interest

The authors declare no conflicts of interest.

## Supporting information




**Supporting File**: advs75132‐sup‐0001‐SuppMat.docx.

## Data Availability

The data that support the findings of this study are available from the corresponding author upon reasonable request.
