# Supplementary material for: Epitaxial Stabilization of Ultrasmall Cu Nanoparticles With High‐Energy {110} Facets on Ti3C2 MXene for Efficient CO2‐to‐Acetate Electrocatalysis
Source: Adv Sci (Weinh). 2026 Apr 2;13(36):e75132. doi: 10.1002/advs.75132 (PMC13317602; doi:10.1002/advs.75132)
Supplement: Supplementary file 1 — Supporting File: advs75132‐sup‐0001‐SuppMat.docx. [file ADVS-13-e75132-s001.docx]

Supporting Information

Epitaxial Stabilization of Ultrasmall Cu Nanoparticles with High-Energy (110) Facets on Ti_3_C_2_ MXene for Efficient CO_2_-to-Acetate Electrocatalysis

Yan-An Li^+[a,b]^, Jiapeng huang^+[a,b]^, Yaohui Zhao ^[a,b]^, Junhao Lu ^[a]^, Yuan Ren ^[a]^, Zi-Xin Ge ^[a,b]^, Qian Wang ^[a]^, Shangdong Ji ^[a]^, Yangzi Zheng ^[a]^, Chao Wu*^*^*^[a,b]^, and Mingshang Jin*^*^*^[a,b]^

*^a^Frontier Institute of Science and Technology* *and State Key Laboratory of Multiphase Flow in Power Engineering,* *Xi’an Jiaotong University, Xi’an, Shaanxi 710049, China.*

*^b^Interdisciplinary Research Center of Frontier Science and Technology, Xi’an Jiaotong University, Xi’an, Shaanxi, 710049, China.*

^+^These authors contributed equally.

*^*^*To whom correspondence should be addressed. E-mail: jinm@mail.xjtu.edu.cn, chaowu@mail.xjtu.edu.cn.

Materials

Hexadecylamine (HDA, CH_3_(CH_2_)_14_CH_2_NH_2_, 99%), D-(+)-glucose (C_6_H_12_O_6_, ≥99.5%), copper chloride dihydrate (CuCl_2_.2H_2_O, 99%), HF (48%), *N, N*-dimethylformamide (DMF, 99%), and Nafion 117 solution (5%) were purchased from Sigma-Aldrich. Aluminum titanium carbide powder (Ti_3_AlC_2_) was purchased from Foshan Xinxi Technology. Potassium hydroxide (KOH, ≥85%), ethanol (C_2_H_5_OH, AR), dimethyl sulfoxide (DMSO), D_2_O, and isopropanol (C_3_H_8_O, AR) were obtained from Aladdin Reagent Co. All the chemicals were used as received. All aqueous solutions were prepared using deionized water with a resistivity of 18.2 MΩ•cm.

Preparation of monolayer Ti_3_C_2_

Two-dimensional Ti_3_C_2_T_x_ nanosheets were prepared by a standard etching method previously reported with slight modifications.^[1,2]^ Mainly, Ti_3_AlC_2_ powder (1 g) was added to an aqueous solution of HF (10 mL, 5 wt%) at 25 ℃ for 24 h under stirring with a magnetic stirrer, followed by consecutive washing and centrifugation cycles of the solid residue at 3500 rpm using deionized water until the supernatant reached a pH of ≥6. Next, the obtained powder was mixed with DMF (10 mL) while stirring for 18 hours, followed by sonication for 2 hours at room temperature to facilitate intercalation with DMF. Afterwards, four centrifugation cycles were performed at 4000 rpm, followed by washing with deionized H_2_O to remove DMF and purify the final supernatant colloidal suspension containing the monolayer Ti_3_C_2_.

Preparation of Cu/Ti_3_C_2_

In a typical synthesis of Cu/Ti_3_C_2_, 10 mL of an aqueous solution containing 45 mg HDA, 12 mg glucose, 5 mg CuCl_2_·2H_2_O, and 0.5 mL of the Ti_3_C_2_ suspension was placed in a 25 mL vial and stirred at room temperature for 12 hours to form a uniform mixture. Then, the mixture was placed in an oil bath and reacted at 100 °C for 1 hour under nitrogen protection with magnetic stirring. The product was collected by centrifugation at 12000 rpm for 5 minutes, washed five times with ethanol, and finally dispersed in 5 mL of ethanol.

Catalyst characterization

Transmission electron microscopy (TEM) images, high-resolution TEM (HRTEM) images, high-angle annular dark-field scanning transmission electron microscopy (HAADF-STEM) images, and energy-dispersive X-ray spectroscopy (EDS) elemental mappings were performed on a JEM-F200 microscope equipped with a built-in EDS operated at 200 kV. Aberration-corrected STEM characterization was conducted on a ThermoFisher Themis Z microscope equipped with two aberration correctors operated at 300 kV. The powder X-ray diffraction (XRD) patterns were collected using a Rigaku SmartLab powder X-ray diffractometer operated at 3 kW, with Cu Kα (λ = 0.15406 nm) radiation. The X-ray photoelectron spectroscopy (XPS) data were acquired using a Thermo Fisher ESCALAB Xi+ spectrometer with monochromatic Al Kα radiation. The Ti 2p, Cu 2p, and O 1s peaks were calibrated using the C 1s contamination peak (284.8 eV) as a reference. The concentrations of Cu and Ti in all samples were measured by inductively coupled plasma mass spectrometry (ICP-MS) using a NexION 350D. The XAFS data were processed in Athena (version 0.9.26) for background subtraction, pre-edge, and post-edge line calibrations. Subsequently, Fourier transform fitting was performed in Artemis (version 0.9.26). For the Cu-foil, the fitting used a k^3^ weighting, a k-range of 3–12.5 Å^–1^, and an R-range of 1–3 Å; the same parameters applied to the sample. Wavelet transform analysis was conducted by importing the χ(k) data from Athena into the Hama Fortran code. The parameters included an R range of 0-4 Å, a k range of 0–16 Å^–1^, a k weight of 2, and a Morlet mother wavelet with κ=10 and σ=1 to analyze the overall distribution.

*In situ* ATR-SEIRAS and *operando* Raman

The *in situ* attenuated total reflection surface-enhanced infrared absorption spectroscopy (ATR-SEIRAS) of electrocatalysts was collected using a Thermo-Fisher Nicolet iS50, equipped with a liquid nitrogen-cooled HgCdTe (MCT) detector and VeeMax III ATR accessory. The sample ink was dropped on a gold film supported by silicon, which served as the working electrode. Ag/AgCl (3.5 M KCl) and a Pt wire were used as the reference electrode and counter electrode, respectively. The electrolyte was a CO_2_-saturated 1 M KOH solution. ATR-SEIRAS measurements were recorded by stepping the potential from -0.1 V to −1.2 V (*vs*. RHE). Spectra collected at open-circuit voltage served as the background. All ATR-SEIRAS measurements involved averaging 64 scans at a spectral resolution of 4 cm^−1^.

*Operando* Raman measurements were performed using a Renishaw inVia Qontor Raman microscope with a 532 nm solid laser as the excitation source. The measurement was conducted in a homemade reactor with a glassy carbon electrode, Ag/AgCl, and a Pt wire as the working electrode, reference electrode, and counter electrode, respectively. The catalyst ink was prepared by mixing isopropanol, water, and Nafion solution. The working electrode was made by drop-casting the catalyst ink onto the glassy carbon electrode. The electrolyte of 1 M KOH was flowed with CO_2_ for 30 minutes before testing. Applied potentials ranged from −0.3 to −1.2 V *vs*. RHE. Raman spectra were collected at different potentials, with each potential applied for at least 5 minutes before spectrum collection to ensure a steady-state condition of the catalyst surface. A Si standard was used to calibrate the *operando* Raman spectroscopy.

Electrochemical measurements

Preparation of the working electrode. Typically, 5 mg of the catalyst and 3 mg of Ketjen black are dispersed in 1 mL of isopropanol and 20 μL of 5% Nafion binder solution under sonication for 1 hour to form a homogeneous ink. Then, 30 μL of the catalyst ink is loaded onto a 1×1 cm² carbon fiber paper electrode and dried at room temperature. The electrochemical CO_2_ reduction reaction measurements are performed in an electrochemical workstation (CHI 760E) using a flow cell with a 1 cm² working area and a 1.0 M KOH electrolyte. A platinum foil (1×1 cm²) and a saturated Ag/AgCl electrode are used as the counter and reference electrodes, respectively. The cathode and anode chambers are separated by a piece of ionic exchange membrane (Fumasep FAA–PK–130, Fuel Cell Stores). During measurements, the electrolyte solution is purged with CO_2_ (99.999%) for 30 minutes to achieve CO_2_ saturation (pH = 7.2). Linear sweep voltammetry (LSV) is performed at a scan rate of 10 mV s^−1^ from 0 V to −0.8 V *vs*. RHE in CO_2_-saturated 1 M KOH electrolyte, conducted in an H cell. All potentials are converted to the reversible hydrogen electrode (RHE) scale using the equation: E (*vs*. RHE) = E (*vs*. Ag/AgCl) + 0.059 × pH + 0.196 V. The CO_2_ gas is supplied at an average rate of 25 mL min^-1^ at room temperature and ambient pressure. The gas phase composition is analyzed by gas chromatography (GC, FuLi GC9790 II) using a thermal conductivity detector (TCD) and a flame ionization detector (FID). The liquid products were analyzed using quantitative proton nuclear magnetic resonance (1H NMR, Agilent, DD2 600 MHz), with DMSO serving as an internal standard. The solvent presaturation technique was applied to suppress the water peak. Specifically, gaseous products such as H_2_, CO, CH_4_, and C_2_H_4_ generated during the electrocatalytic process were detected by online GC equipped with TCD and FID, using Ar (99.999%) as the carrier gas. Liquid products like HCOOH, C_2_H_5_OH, and CH_3_COOH formed during the electrocatalytic process were analyzed with 1H NMR. Briefly, after the electrocatalytic CO_2_RR tests, 0.1 mL of catholyte was mixed with 0.1 mL DMSO (0.5 mM) solution as an internal standard and 0.3 mL D_2_O for identification and quantification of the liquid products. The area ratios of the peaks for HCOOH, C_2_H_5_OH, and CH_3_COOH relative to the DMSO peak were compared to the standard curve to determine their concentrations, as shown in Figure S12. The Faradaic efficiency (FE) was calculated using the following equation.^[3]^

The following equation calculated the Faradic efficiency of the gas products.

$$\mathbf{FE}_{\mathbf{gas}}\mathbf{=}\frac{\mathbf{C}_{\boldsymbol{x}}\boldsymbol{\times V\times P\times\alpha\times F}}{\boldsymbol{R\times T\times Q}}\boldsymbol{\times100\%}$$

where, C_x_ is the volumetric concentration of the gas products; V represents the total gas volume of electrolytic cell; P is atmospheric pressure, 101,325 Pa N m^−2^; α is the electron transfer number for specific gas products; F is Faraday's constant, 96,485 C mol^−1^; R is the gas constant, 8.314 J mol^−1^ K^−1^; T is 298.15 K; Q is the total electricity passed through the system.

The following equation calculated the Faradic efficiency of the liquid products.

$$\mathbf{FE}_{\mathbf{liquid}}\mathbf{=}\frac{\mathbf{n}_{\mathbf{product}}\boldsymbol{\times\alpha\times F}}{\mathbf{Q}}\boldsymbol{\times100\%}$$

where n_product_ represents the concentration of liquid products in the electrolyte (mol); α is the electron transfer number for specific gas products; F is Faraday's constant, 96,485 C mol^−1^, and Q is the total electricity passed through the system.

Computational details

Density functional theory (DFT) calculations are performed using the Vienna Ab Initio Simulation Package (VASP) with the projector augmented wave method.^[4]^ The generalized gradient approximation (GGA), specifically the Perdew-Burke-Ernzerhof (PBE) functional, is employed to handle the exchange-correlation functional, with a plane-wave energy cutoff of 450 eV. The van der Waals weak interaction was described using the DFT-D3 method.^[5]^ The atoms in the bottom layer are fixed during surface relaxation. In contrast, the atomic positions of other layers are fully relaxed until the energies and forces on all atoms are less than 10^−5^ eV and 0.02 eV Å^-1^, respectively. A 15 Å vacuum layer was added to minimize interactions between its images. The Brillouin zone integration is performed using a Monkhorst−Pack grid of 6 × 6 × 1, 4 × 6 × 1, and 4 × 2 × 1 k-points for Cu (100), Cu (110), and Ti_2_C_3_ (100) slab models, respectively.^[6,7]^

The adsorption energy $\boldsymbol{E}_{\mathbf{ads}}$ is defined as:

$\boldsymbol{E}_{\mathbf{ads}}\mathbf{=}\boldsymbol{E}_{\mathbf{total}}\mathbf{-}\boldsymbol{E}_{\mathbf{slab}}\mathbf{-}\boldsymbol{E}_{\mathbf{adsorbate}}$

where$\boldsymbol{E}_{\mathbf{total}}$ is the total energy of the adsorbed system, $\boldsymbol{E}_{\mathbf{slab}}$ is the energy of the clean slab, and $\boldsymbol{E}_{\mathbf{adsorbate}}$ is the total energy of the isolated adsorbate.

The Climbing Image Nudged Elastic Band (CI-NEB) methods were used to search for the transition states (TS). The energy barrier is defined as follows:

$$\boldsymbol{E}_{\boldsymbol{barrier}}\mathbf{=}\boldsymbol{E}_{\boldsymbol{TS}}\mathbf{-}\boldsymbol{E}_{\boldsymbol{IS}}$$

where $\boldsymbol{E}_{\boldsymbol{TS}}$ is the total energy of the transition state, $\boldsymbol{E}_{\boldsymbol{IS}}$ is the energy of the initial state.

The Gibbs free energy (ΔG) was calculated by the following equation:

$$\boldsymbol{\Delta}\boldsymbol{G}\boldsymbol{= \Delta}\boldsymbol{E}\boldsymbol{+ \Delta}\boldsymbol{E}_{\mathbf{ZPE}}\mathbf{-}\boldsymbol{T}\boldsymbol{\Delta}\boldsymbol{S}$$

where ΔE represents the reaction energy difference between the reactant and product, which can be directly obtained from the DFT results. $\boldsymbol{\Delta}\boldsymbol{E}_{\mathbf{ZPE}}$ and $\boldsymbol{\Delta}\boldsymbol{S}$ are the change in the zero-point energy and entropy at room temperature (T = 298.15 K), which can be computed from the vibrational frequencies via using the VASPKIT package.


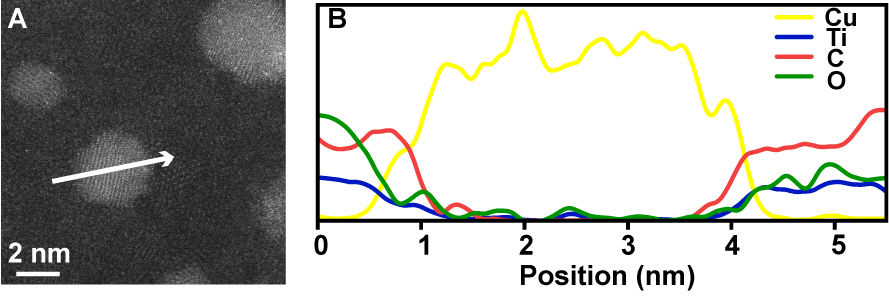


Figure S1. (A) HAADF-STEM images of Cu/Ti_3_C_2_. (B) Compositional line profile of Cu/Ti_3_C_2_ catalyst recorded along the arrow in the STEM image.


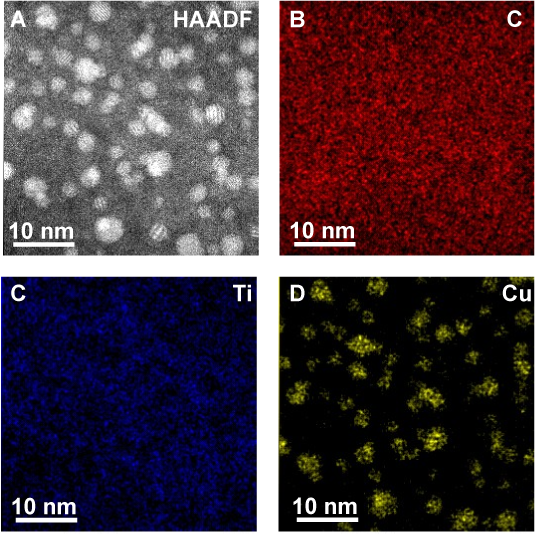


Figure S2. (A-D) HAADF-STEM images and corresponding EDS mappings of the Cu/Ti_3_C_2_.


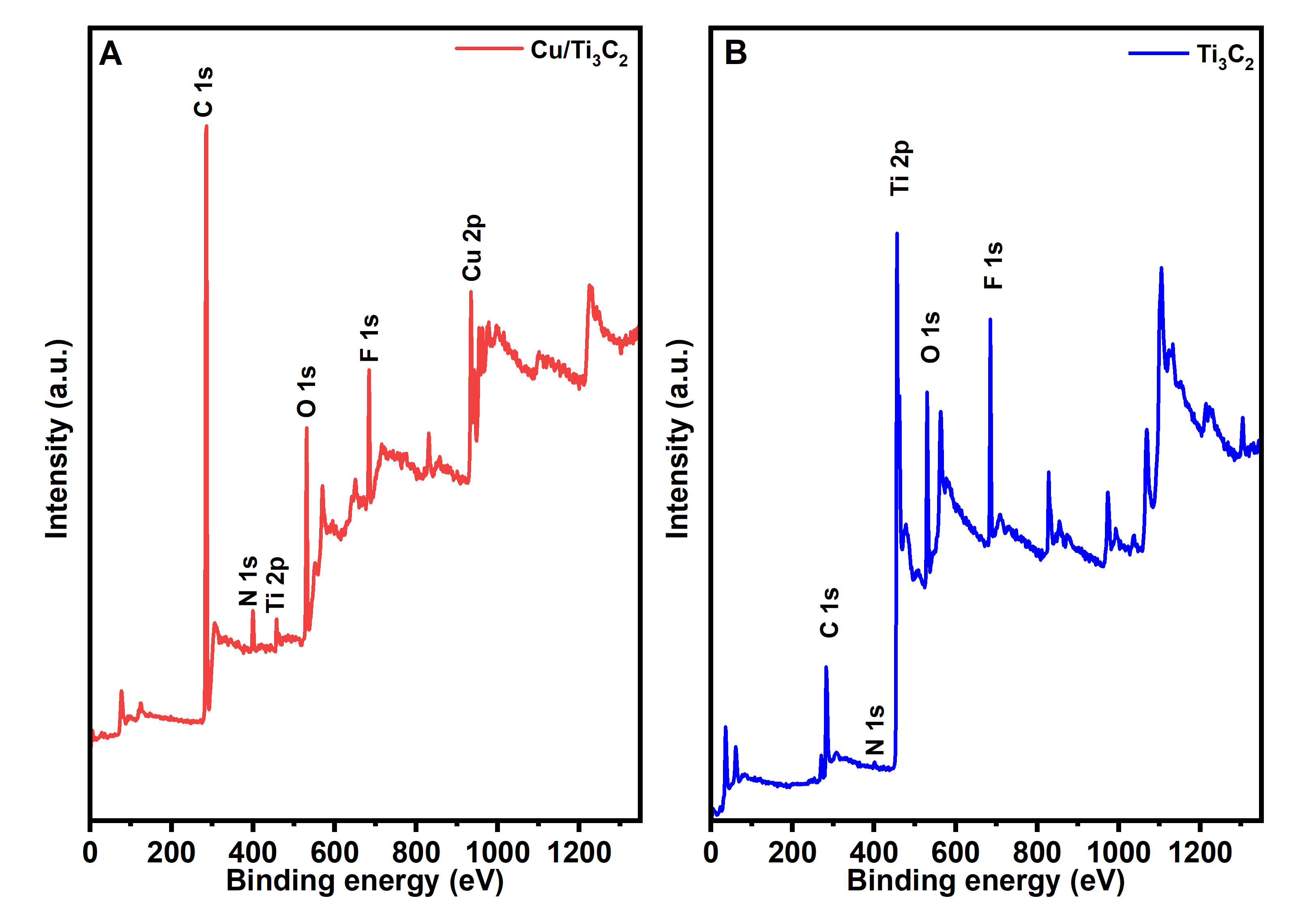


Figure S3. Survey X-ray photoelectron spectra (XPS) of Cu/Ti_3_C_2_ catalyst and Ti_3_C_2_ support.


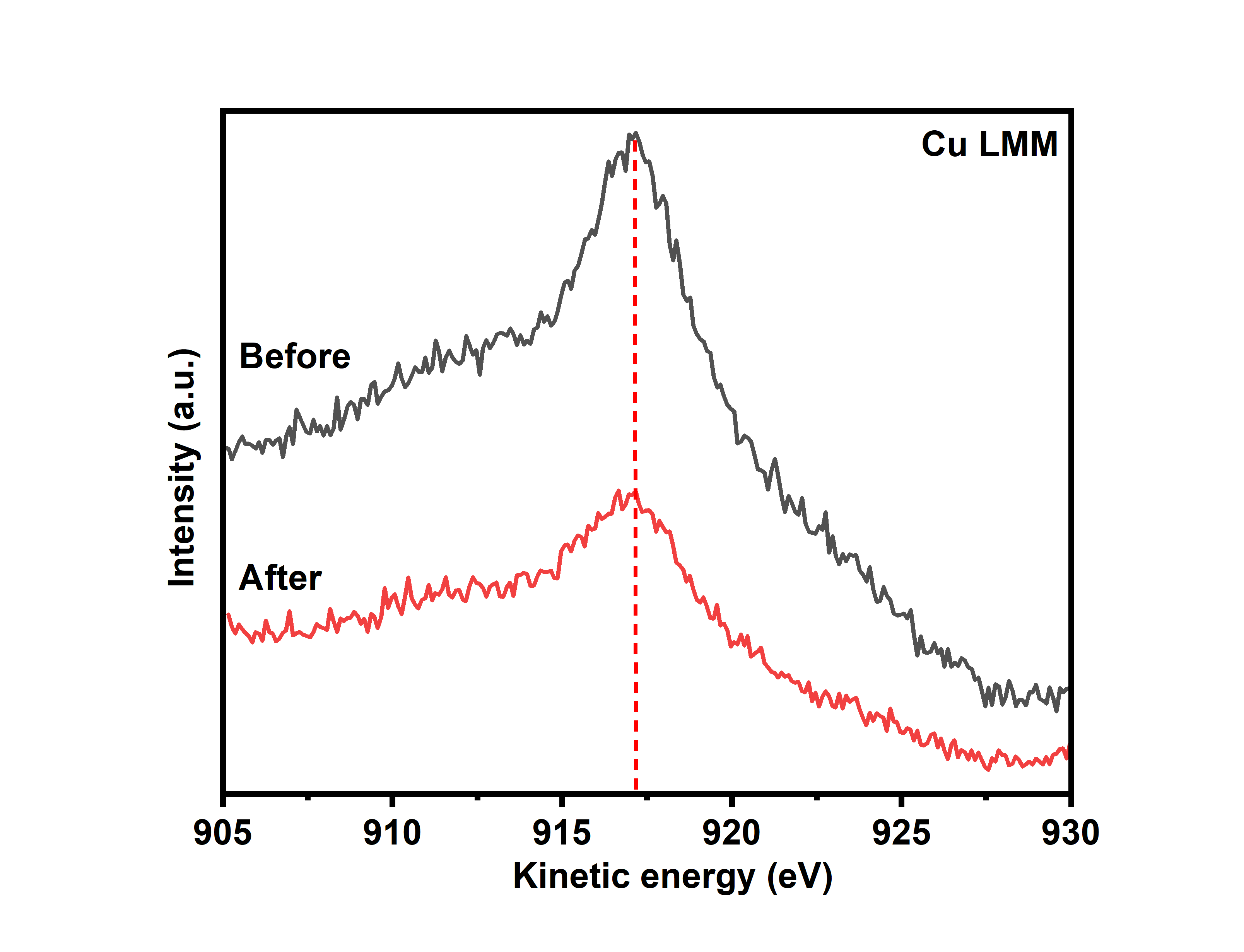


Figure S4. XPS spectra of Cu LMM in Cu/Ti_3_C_2_ catalyst before and after stability test.


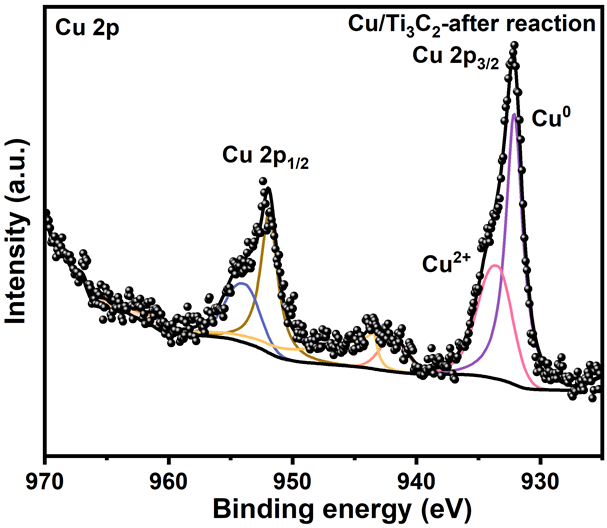


Figure S5. XPS spectra of Cu 2p in Cu/Ti_3_C_2_ catalyst after stability test.


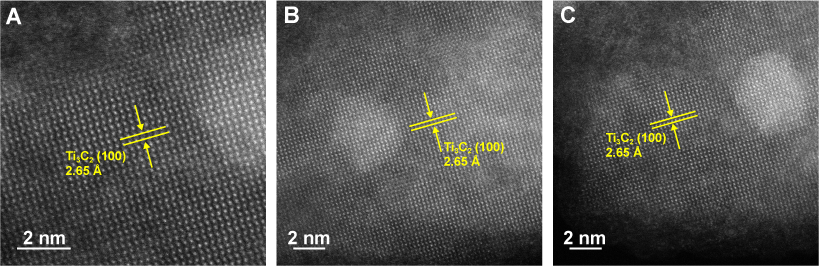


Figure S6.AC-HAADF-STEM images of Ti_3_C_2_ support.


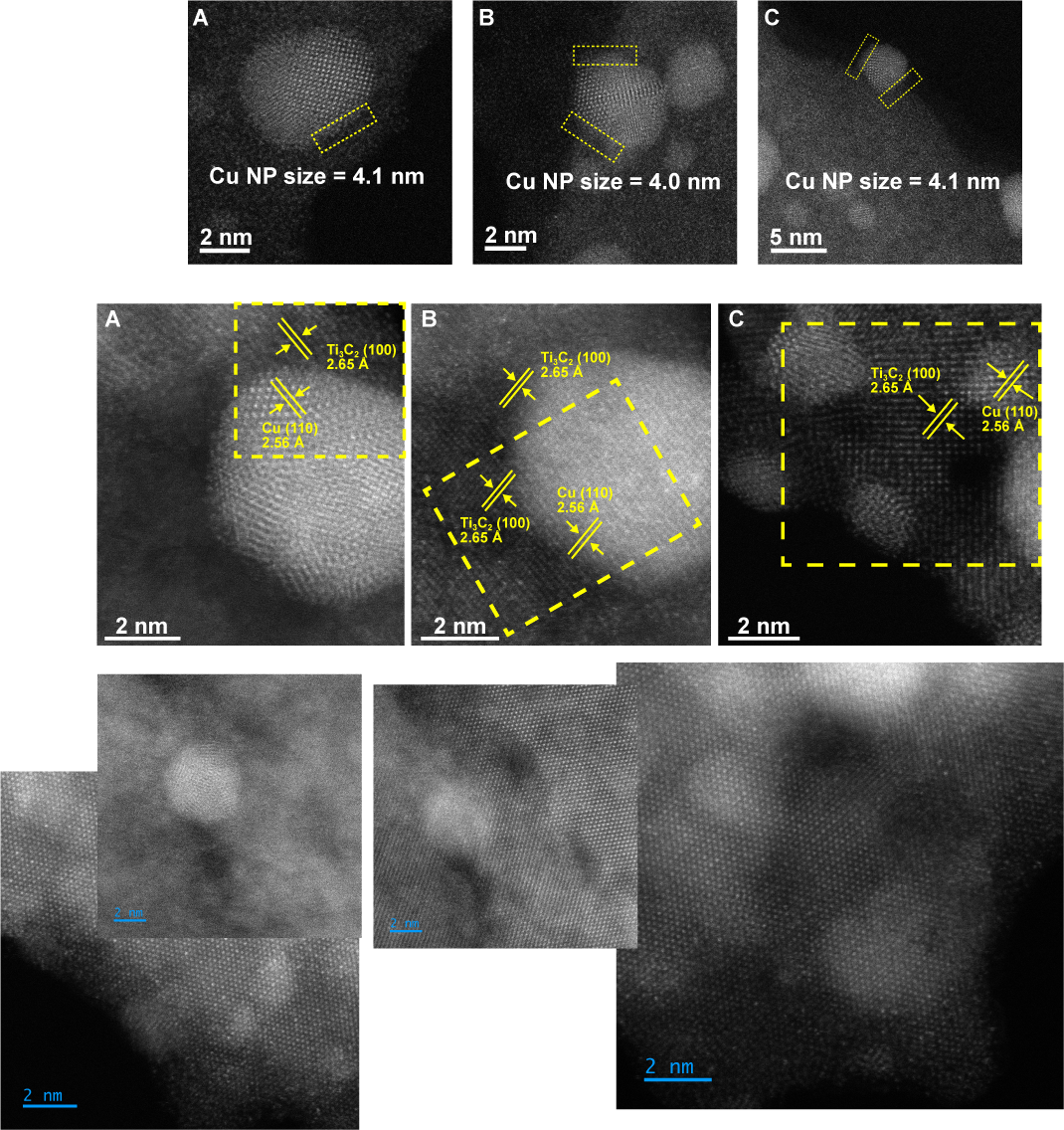


Figure S7. AC-HAADF-STEM images of the Cu/Ti_3_C_2_ interfacial region.


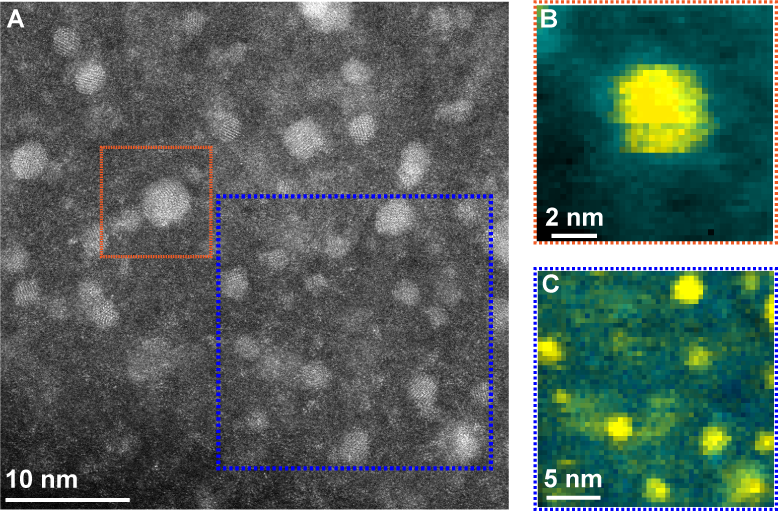


Figure S8. (A) HAADF-STEM images of Cu/Ti_3_C_2_, dotted-line rectangles show the areas where electron energy-loss spectrum images were acquired (EELS). (B, C) EELS maps for the different Cu elements valence states are shown (Cu^2+^/blue, Cu^0^/yellow).

Electron energy-loss spectroscopy (EELS), now routinely integrated into transmission electron microscopes, has become a powerful tool for analyzing nanocatalyst materials. By providing element-specific information at sub-nanometer resolution, EELS simultaneously maps valence states, measures compositional gradients, and uncovers structural correlations that are now well established for atomic-level studies of electrochemical nanocatalysts. The valence states of Cu in Cu/Ti_3_C_2_ were confirmed by EELS. As shown in Figure S7, the distribution of valence (Cu^2+^/Cu^0^) displays a distinct profile, offering definitive evidence that the valence state of Cu nanoparticles in Cu/Ti_3_C_2_ is 0.^[8-11]^


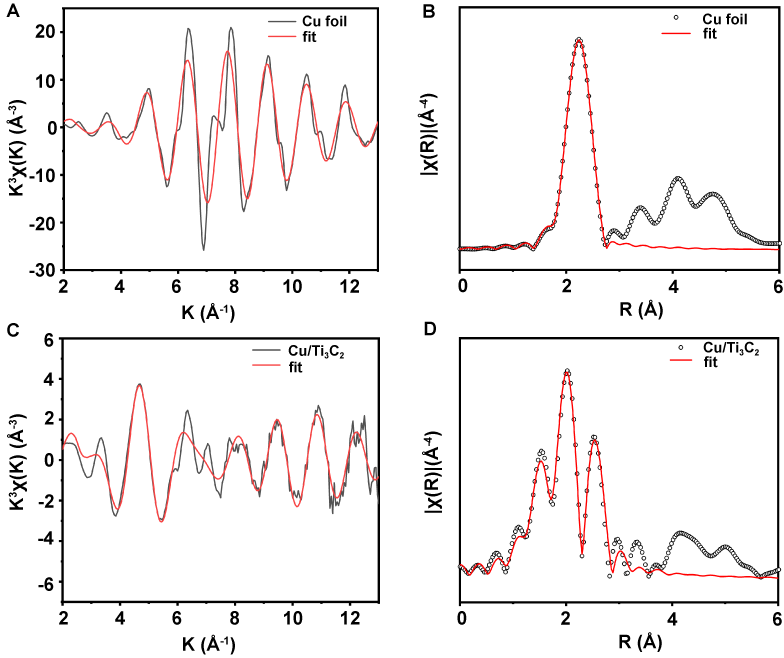


Figure S9. Fitted EXAFS spectra of different catalysts in K space and R space.


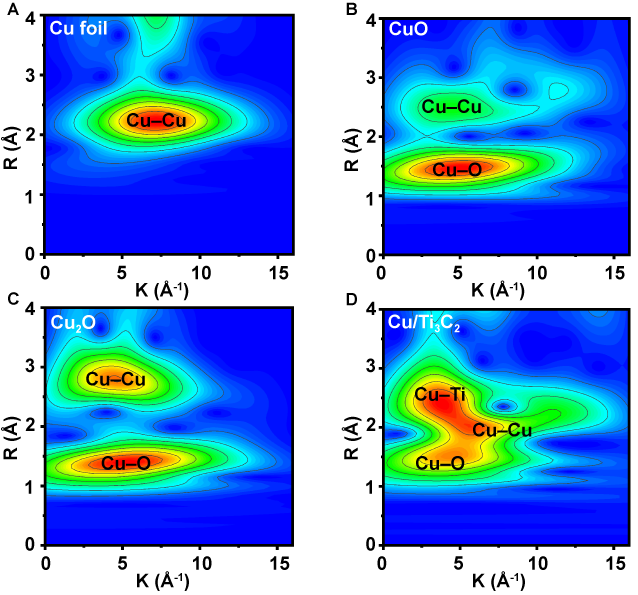


Figure S10. Wavelet transform analysis of the first coordination shell for Cu foil, CuO, Cu_2_O, and Cu/Ti_3_C_2_.


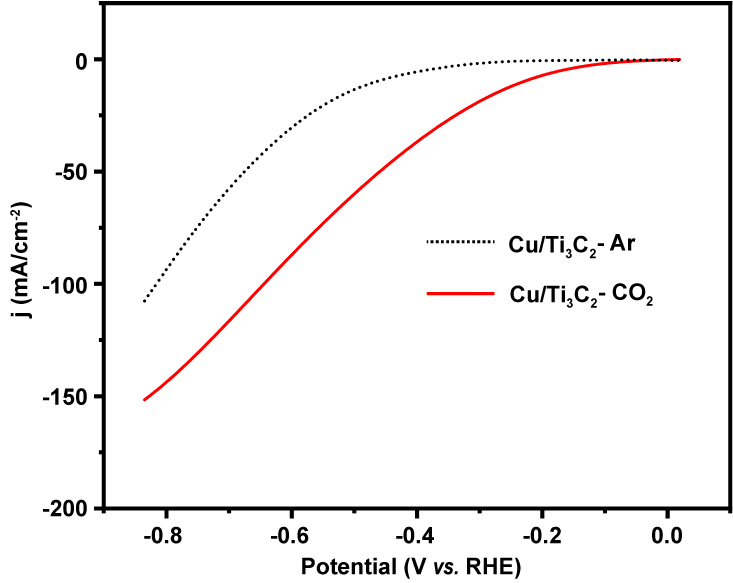


Figure S11. LSV curves of Cu/Ti_3_C_2_ in CO_2_/Ar saturated electrolyte (LSV measurement conducted in H cell).


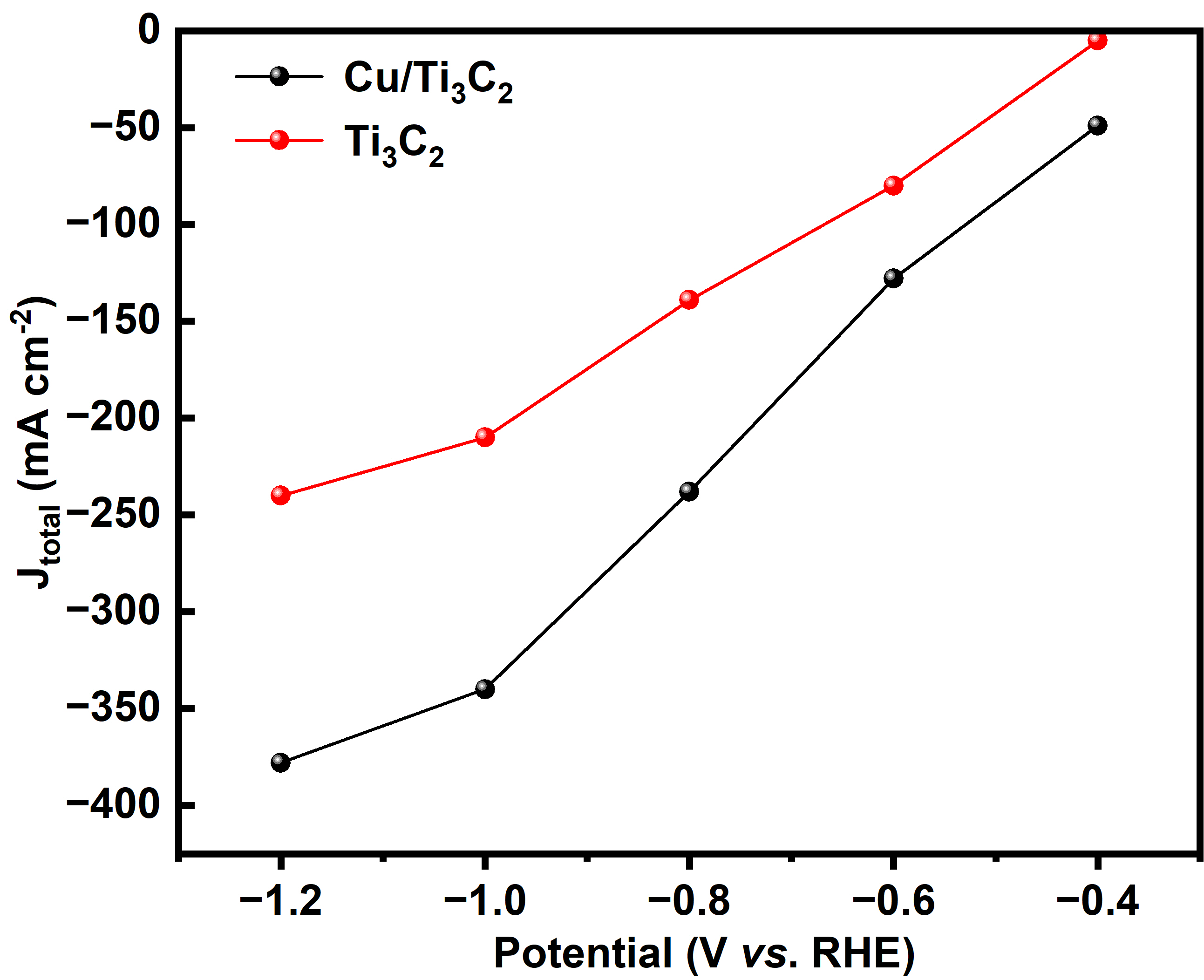


Figure S12. Total current densities at different potentials for Cu/Ti_3_C_2_ and Ti_3_C_2_.


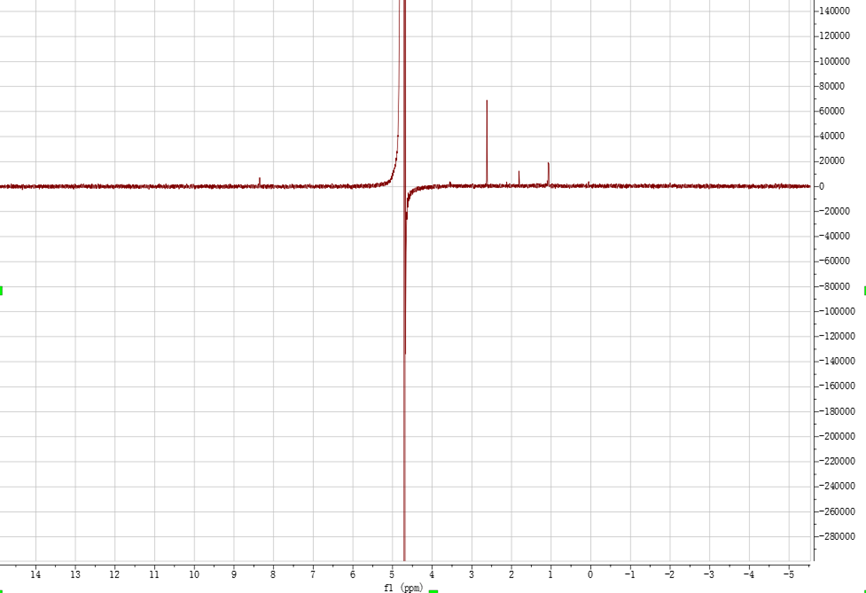


Figure S13. Representative ^1^H-NMR (600 MHz, D_2_O) spectrum taken on the liquid products in a flow cell.


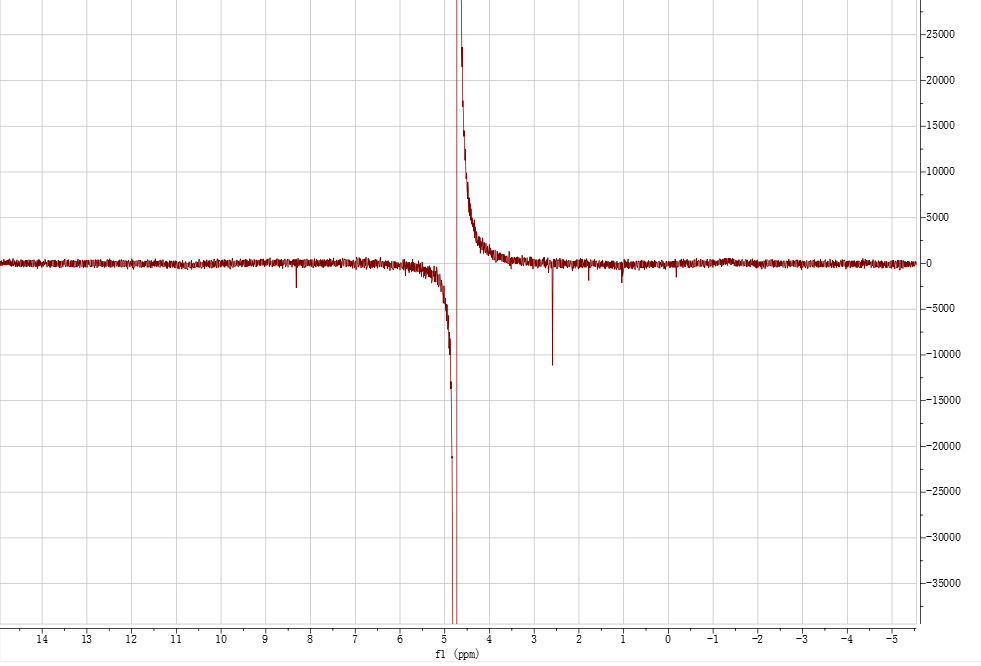


Figure S14. Representative ^1^H-NMR (600 MHz, D_2_O) spectrum taken on the liquid products in a H-cell.


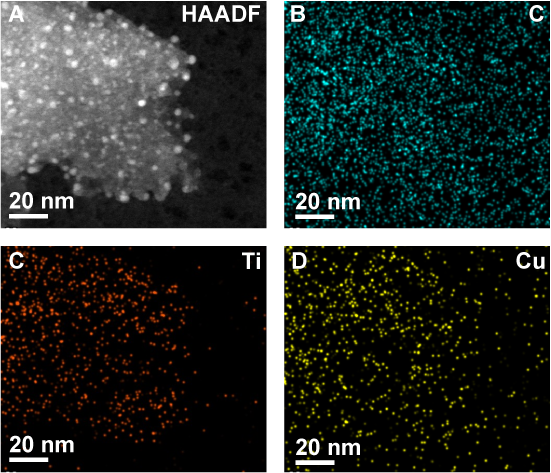


Figure S15. (A-D) HAADF-STEM images and corresponding EDS mappings of the Cu/Ti_3_C_2_ after stability test.


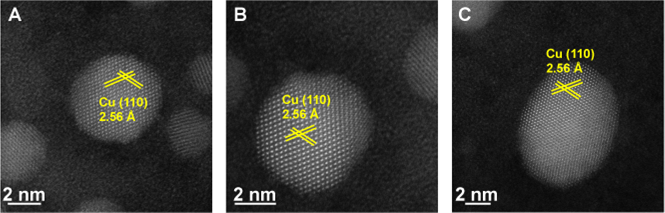


Figure S16. AC-HAADF-STEM images of Cu NPs after stability test.


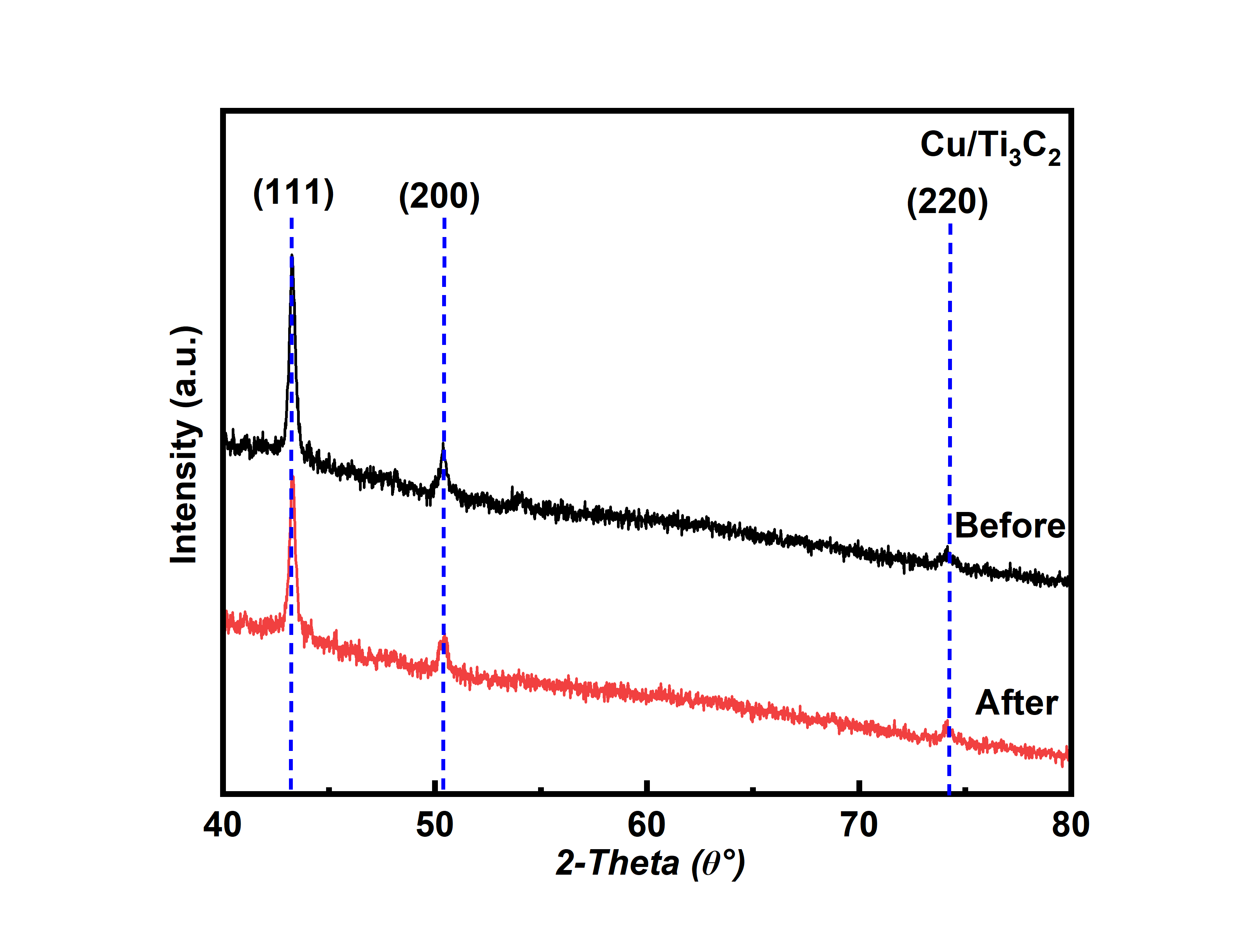


Figure S17. XRD patterns of Cu/Ti_3_C_2_ catalyst before and after stability test.


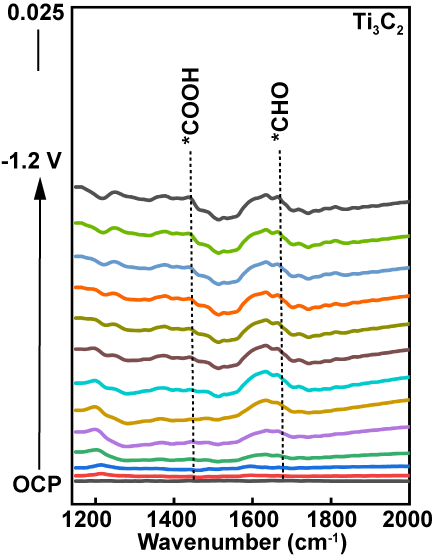


Figure S18. *In situ* ATR-SEIRAS spectra recorded by ramping down the potential from -0.1 to -1.2 V to monitor the intermediates evolution on Ti_3_C_2_ in electrocatalytic CO_2_RR.


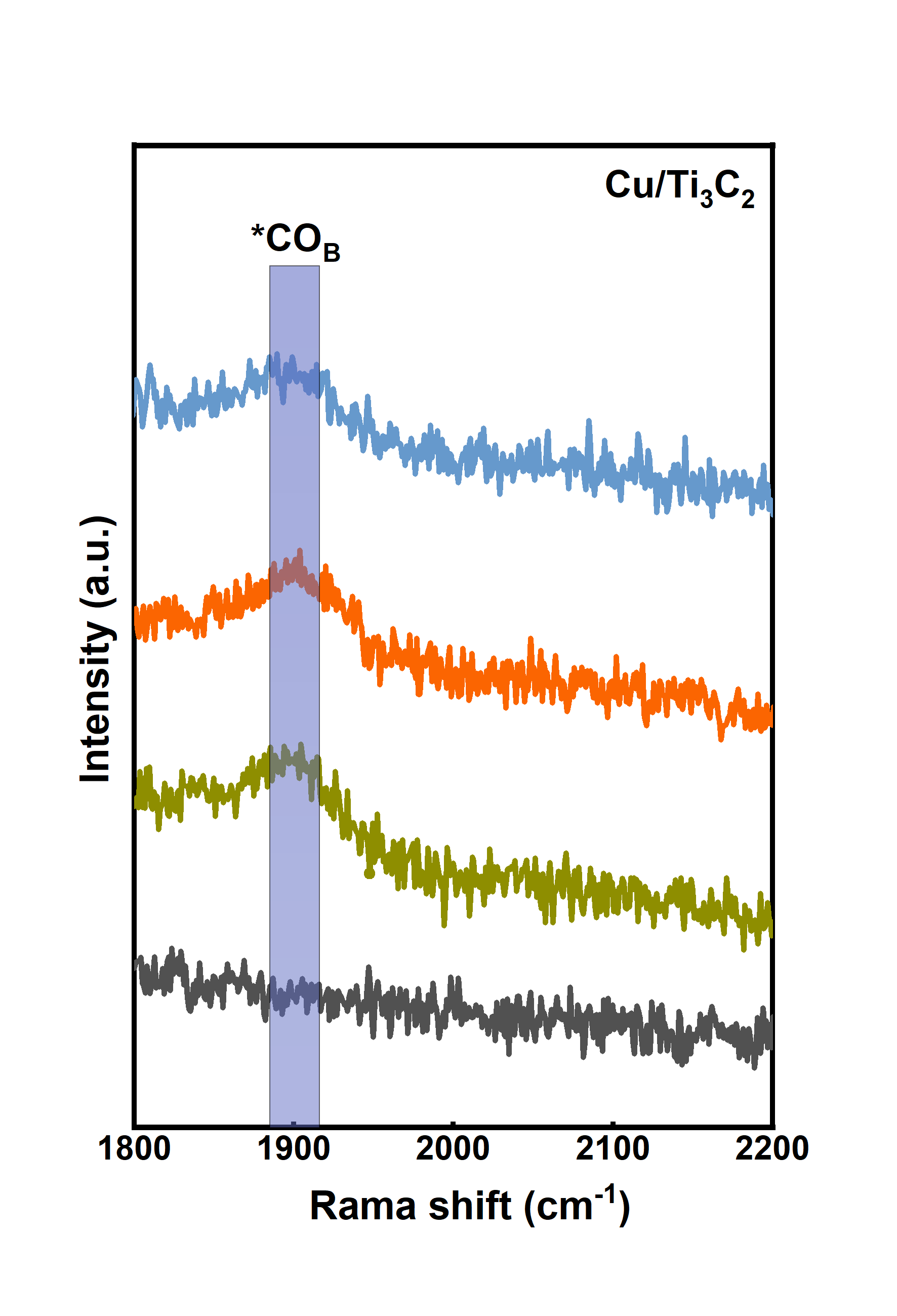


Figure S19. *Operando* Raman spectra to monitor the intermediates evolution on Cu/Ti_3_C_2_ in electrocatalytic CO_2_RR.


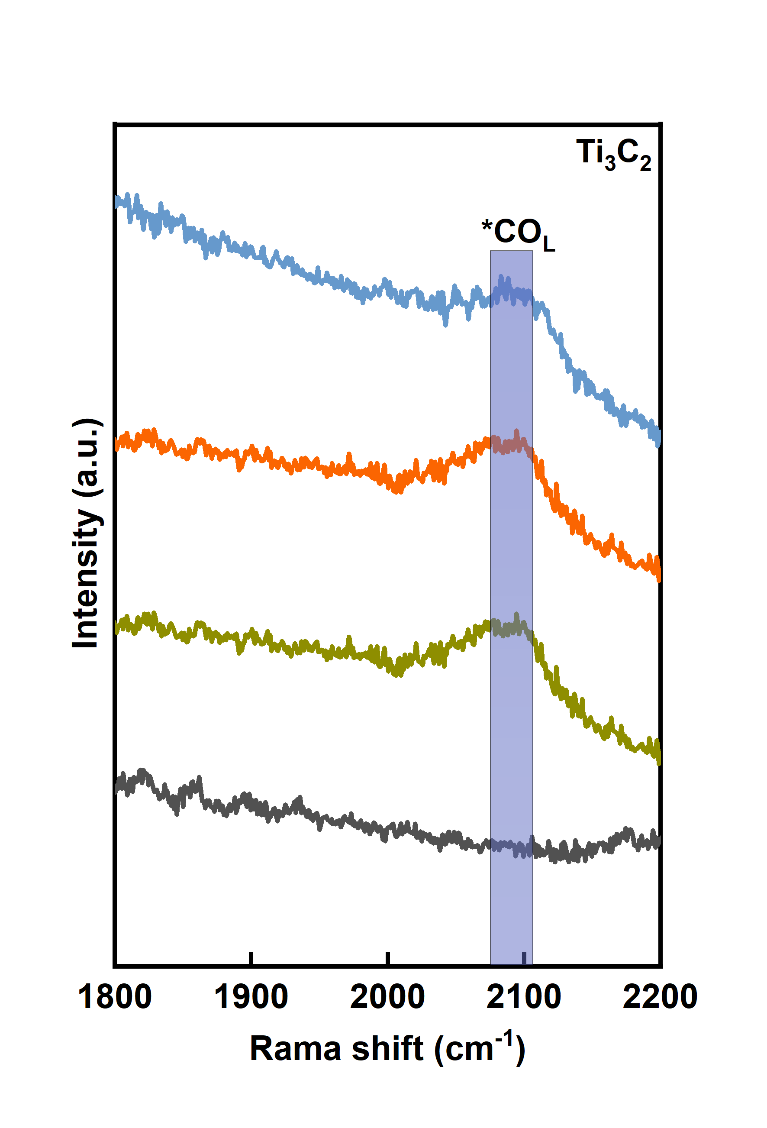


Figure S20. *Operando* Raman spectra to monitor the intermediates evolution on Ti_3_C_2_ in electrocatalytic CO_2_RR.


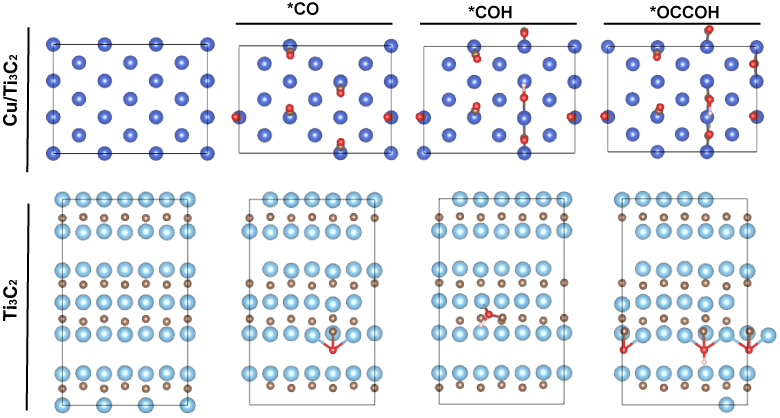


Figure S21. DFT calculation models. Configurations of *CO, *COH, and *OCCOH intermediates adsorbed on the Cu/Ti_3_C_2_ and Ti_3_C_2_ surfaces.


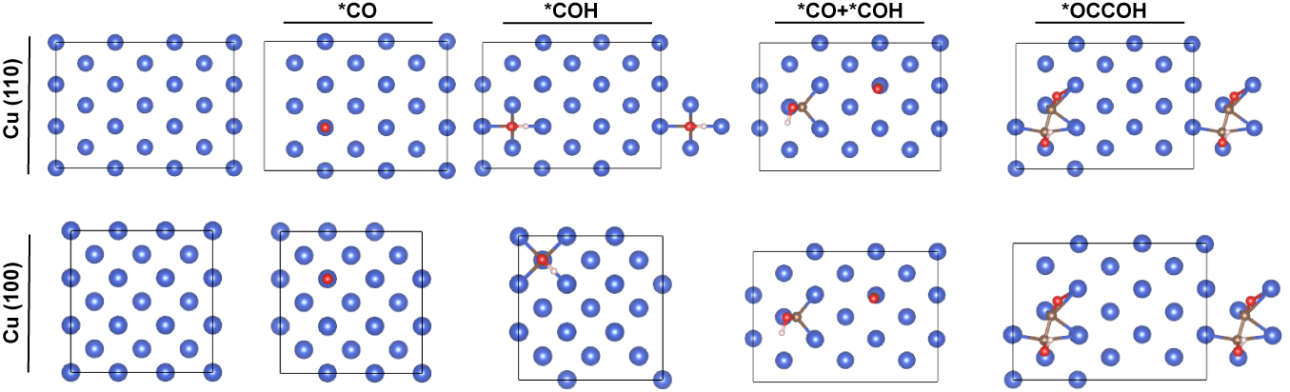


Figure S22. DFT calculation models. Configurations of *CO, *COH, *CO + *COH, and *OCCOH intermediates adsorbed on the Cu(110) crystal facet and Cu(100) crystal facet.

Table S1. The hkl indices, the FWHM values, and the D values (estimated from the Scherrer equation) of the Cu/Ti_3_C_2_.

| Sample | hkl | FWHM | D/nm |
| --- | --- | --- | --- |
| Cu/Ti_3_C_2_ | 111 | 2.105 | 4.02 |
| Cu/Ti_3_C_2_ | 200 | 2.048 | 4.13 |
| Cu/Ti_3_C_2_ | 220 | 2.228 | 4.17 |

Table S2. Cu K-edge EXAFS curve fitting parameters.

| Sample | Path | CN | R(Å) | σ^2^(Å^2^) | ΔE_0_(eV) | R factor |
| --- | --- | --- | --- | --- | --- | --- |
| Cu-foil | Cu-Cu | 12 | 2.54±0.01 | 0.0086 | 4.4±0.5 | 0.0030 |
| Cu/Ti_3_C_2_ | Cu-O | 0.7±0.1 | 1.89±0.01 | 0.0058 | -1.5±0.9 | 0.0166 |
|  | Cu-Cu | 3.7±0.2 | 2.50±0.01 | 0.0080 |  |  |
|  | Cu-Ti | 3.6±0.2 | 2.76±0.01 | 0.0073 |  |  |

Ѕ_0_^2^: the amplitude reduction factor (Ѕ_0_^2^=0.87 from Cu-foil); CN: coordination numbers; R: bond distance; σ^2^: Debye-Waller factors; ΔE_0_: the inner potential correction. R factor: goodness of fit.

Table S3. Comparison of this work to the prior reports on the CO_2_ electroreduction

performance.

| Electrocatalyst | Potential (V) | Current density (mA cm^-2^) | Electrolyte | Faradaic efficiency (% acetic acid) | Faradaic efficiency (% acetate) | Reference |
| --- | --- | --- | --- | --- | --- | --- |
| Cu/Ti_3_C_2_ | -0.8 (*vs*. RHE) | 235 | 1 M KOH | 42.5 | - | This work |
| Cu(I) complex/BN-C_30_ | −1.3 (*vs*. RHE) | 13.9 | BF_4_-LiI-water solution | 80.3 | - | [12] |
| Pd-MWNTs | - | 2.13 | 0.5 M KHCO_3_ | - | 52.3 | [13] |
| SiW_9_V_3_ | −1.15 (*vs*. Ag/AgCl) | 1 | 0.1 M Na_2_SO_4_ | - | 96.5 | [14] |
| NDD/Si | −1.0 (*vs*. RHE) | 2 | 0.5 M NaHCO_3_ | - | 77 | [15] |
| Ni SACs-Cu NPs | −0.5 (*vs*. RHE) | 50 | - 0.1 M KHCO_3_ | - | 45 | [16] |
| COF | −0.8 (*vs*. RHE) | 12.5 | 0.1 M KHCO_3_ | - | 90.3 | [17] |
| Cu_2_O-CuO | −0.4 (*vs*. RHE) | 0.46 | 0.3 M KHCO_3_ | 84 | - | [18] |
| PAn | −0.4 (*vs*. RHE) | 1.2 | MeOH  + LiClO_4_ + H + H_2_O | 57 | - | [19] |
| CuPd-1.5 | −0.7 (*vs*. RHE) | 25 | 0.5 M KHCO_3_ | 46.5 | - | [20] |
| MnCor-CP | −1.25 (*vs*. Ag/AgCl) | 1 | 0.1 M Phosphate buffer | 63 | - | [21] |
| Ce(OH)x/Cu | −0.7 (*vs*. RHE) | 250 | 1 M KOH | 5 | - | [22] |

Table S4. Comparison of product selectivity with different supports on the CO_2_ electroreduction performance.

| Sample | Metal | Supported | Main product | References |
| --- | --- | --- | --- | --- |
| Cu/SiO_2_ | Cu | SiO_2_ | Ethylene | [23] |
| Cu_cube_/BSi | Cu | SiO_2_ | Methane | [24] |
| Cu_cube_/GC | Cu | Glassy carbon | Ethylene | [24] |
| Cu/RGO | Cu | Reduced graphene oxide | Formic Acid | [25] |
| Cu/CNT | Cu | Carbon nanotubes | Carbon monoxide | [25] |
| Cu/CB | Cu | Carbon black | Carbon monoxide | [25] |
| Cu-Ti_3_C_2_T_X_ | Cu | Ti_3_C_2_T_X_ | Carbon monoxide | [26] |
| Cu/Ti_3_C_2_ | Cu | Ti_3_C_2_ | Methanol | [27] |

Table S5. The content of Cu and Ti in Cu/Ti_3_C_2_ before and after electrocatalytic CO_2_RR samples was analyzed by ICP-MS.

| Cu/Ti_3_C_2_ | Before | After |
| --- | --- | --- |
| Cu concentration  (10^-3^ g/mL) | 0.168 | 0.159 |
| Ti concentration  (10^-3^ g/mL) | 0.127 | 0.121 |

Reference

[1] K. Eid, Q. Lu, S. Abdel-Azeim, A. Soliman, A. M. Abdullah, A. M. Abdelgwad, R. P. Forbes, K. I. Ozoemena, R. S. Varma, M. F. Shibl, Highly exfoliated Ti_3_C_2_Tx MXene nanosheets atomically doped with Cu for efficient electrochemical CO_2_ reduction: An experimental and theoretical study, *J. Mater. Chem. A* 2022, *10*, 1965–1975.

[2] M. Alhabeb, K. Maleski, B. Anasori, P. Lelyukh, L. Clark, S. Sin, Y. Gogotsi, Guidelines for synthesis and processing of two-dimensional titanium carbide (Ti_3_C_2_Tx MXene), *Chem. Mater.* 2017, *29*, 7633–7644.

[3] Y. Wang, H. Xu, Y. Liu, J. Jang, X. Qiu, E. P. Delmo, Q. Zhao, P. Gao, M. Shao, A sulfur-doped copper catalyst with efficient electrocatalytic formate generation during the electrochemical carbon dioxide reduction reaction, *Angew. Chem. Int. Ed.* 2024, *136*, e202313858.

[4] G. Kresse, J. Furthmüller, Efficient iterative schemes for ab initio total-energy calculations using a plane-wave basis set, *Phys. Rev. B* *Condens. Matter* 1996, *54*, 11169-11186.

[5] P. E. Blöchl, Projector augmented-wave method, *Phys. Rev. B* *Condens. Matter* 1994, *50*, 17953-17979.

[6] J. P. Perdew, K. Burke, M. Ernzerhof, Perdew, burke, and ernzerhof reply, *Phys. Rev. Lett.* 1998, *80*, 891.

[7] J. K. Nørskov, J. Rossmeisl, A. Logadottir, L. Lindqvist, J. R. Kitchin, T. Bligaard, H. Jónsson, Origin of the overpotential for oxygen reduction at a fuel-cell cathode, *J. Phys. Chem. B* 2004, *108*, 17886-17892.

[8] Z. Yin, W. Zhao, J. Li, X. Peng, C. Lin, M. Zhang, Z. Zeng, H. Liao, H. Chen, H. Lin, F. Pan, Advanced electron energy loss spectroscopy for battery studies, *Adv. Funct. Mater.* 2022, *32*, 2107190.

[9] R. Shi, Q. Li, X. Xu, B. Han, R. Zhu, F. Liu, R. Qi, X. Zhang, J. Du, J. Chen, D. Yu, X. Zhu, J. Guo, P. Gao, Atomic-scale observation of localized phonons at FeSe/SrTiO_3_ interface, *Nat. Commun.* 2024, *15*, 3418.

[10] S. Li, G. Wang, H. Lv, Z. Lin, J. Liang, X. Liu, Y. G. Wang, Y. Huang, G. Wang, Q. Li, Constructing gradient orbital coupling to induce reactive metal–support interaction in Pt-carbide electrocatalysts for efficient methanol oxidation, *J. Am. Chem. Soc.* 2024, *146*, 17659–17668.

[11] T. W. van Deelen, C. Hernández Mejía, K. P. de Jong, Control of metal-support interactions in heterogeneous catalysts to enhance activity and selectivity, *Nat. Catal.* 2019, *2*, 955–970.

[12] X. Sun, Q. Zhu, X. Kang, H. Liu, Q. Qian, J. Ma, Z. Zhang, G. Yang, B. Han, Design of a Cu(I)/C-doped boron nitride electrocatalyst for efficient conversion of CO_2_ into acetic acid, *Green Chem.* 2017, *19*, 2086–2091.

[13] G. Lu, H. Wang, Z. Bian, X. Liu, Electrochemical reduction of CO_2_ to organic acids by a Pd-MWNTs gas-diffusion electrode in aqueous medium, *Sci. World J.* 2013, *2013*, 424617.

[14] B. Zha, C. Li, J. Li, Efficient electrochemical reduction of CO_2_ into formate and acetate in polyoxometalate catholyte with indium catalyst, *J. Catal.* 2020, *382*, 69–76.

[15] Y. Liu, S. Chen, X. Quan, H. Yu, Efficient electrochemical reduction of carbon dioxide to acetate on nitrogen-doped nanodiamond, *J. Am. Chem. Soc.* 2015, *137*, 11631–11636.

[16] X. Hu, J. Li, Z. Zhou, L. Wen, Tandem electroreduction of CO_2_ to programmable acetate and syngas via single-nickel-atom-encapsulated copper nanocatalysts, *ACS Mater. Lett.* 2023, *5*, 85–94.

[17] X. Qiu, J. Huang, C. Yu, Z. Zhao, H. Zhu, Z. Ke, P. Liao, X. Chen, A stable and conductive covalent organic framework with isolated active sites for highly selective electroreduction of carbon dioxide to acetate, *Angew. Chem. Int. Ed.* 2022, *61*, e202206470.

[18] M. Serafini, F. Mariani, A. Fasolini, E. Scavetta, F. Basile, D. Tonelli, Nanostructured copper-based electrodes electrochemically synthesized on a carbonaceous gas diffusion membrane with catalytic activity for the electroreduction of CO_2_, *ACS Appl. Mater. Interfaces* 2021, *13*, 57451–57461.

[19] R. Aydin, F. Köleli, Electrochemical reduction of CO_2_ on a polyaniline electrode under ambient conditions and at high pressure in methanol, *J. Electroanal. Chem.* 2002, *535*, 107–112.

[20] S. Nie, L. Wu, Q. Liu, X. Wang, Entropy-derived synthesis of the CuPd sub-1nm alloy for CO_2_-to-acetate electroreduction, *J. Am. Chem. Soc.* 2024, *146*, 29364-29372.

[21] R. De, S. Gonglach, S. Paul, M. Haas, S. S. Sreejith, P. Gerschel, U. P. Apfel, T. H. Vuong, J. Rabeah, S. Roy, W. Schöfberger, Electrocatalytic reduction of CO_2_ to acetic acid by a molecular manganese corrole complex, *Angew. Chem. Int. Ed.* 2020, *132*, 10614–10621.

[22] M. Luo, Z. Wang, Y. C. Li, J. Li, F. Li, Y. Lum, D. H. Nam, B. Chen, J. Wicks, A. Xu, T. Zhuang, W. R. Leow, X. Wang, C. Dinh, Y. Wang, Y. Wang, D. Sinton, E. H. Sargent, Hydroxide promotes carbon dioxide electroreduction to ethanol on copper via tuning of adsorbed hydrogen, *Nat. Commun.* 2019, *10*, 5814.

[23] S. Yan, S. Gong, S. Zhang, H. Sun, H. Yu, L. Chen, J. Han, H. Wang, In situ/operando insights into the selectivity of CH_4_/C_2_H_4_ in CO_2_ electroreduction by fine-tuning the composition of Cu/SiO_2_ catalysts, *ChemSusChem* 2025, 18, e202402461.

[24] J. Wang, T. Y. Lai, H. T. Lin, T.-R. Kuo, H. C. Chen, C. S. Tseng, C. W. Tung, C. Y. Chien, H. M. Chen, Light-induced dynamic activation of copper/silicon interface for highly selective carbon dioxide reduction, *Angew. Chem. Int. Ed.* 2024, 63, e202403333.

[25] C. Jiménez, M. I. Cerrillo, F. Martínez, R. Camarillo, J. Rincón, Effect of carbon support on the catalytic activity of copper-based catalyst in CO_2_ electroreduction, *Sep. Purif. Technol.* 2020, 248, 117083.

[26] Q. Zhao, C. Zhang, R. Hu, Z. Du, J. Gu, Y. Cui, X. Chen, W. Xu, Z. Cheng, S. Li, B. Li, Y. Liu, W. Chen, C. Liu, J. Shang, L. Song, S. Yang, Selective etching quaternary MAX phase toward single atom copper immobilized MXene (Ti_3_C_2_Clx) for efficient CO_2_ electroreduction to methanol, *ACS Nano* 2021, 15, 4927–4936.

[27] L. Liu, H. Li, T. Yuan, J. Zhang, K. Xue, J. Hou, G. Cao, Copper anchored MXene regulated metal-oxide interfaces for the CO_2_ electrocatalytic conversion, *J. Mater. Sci. Technol.* 2026, 250, 17–24.
